# BEATRICE: Bayesian Fine-mapping from Summary Data using Deep Variational Inference

**DOI:** 10.1101/2023.03.24.534116

**Published:** 2023-03-25

**Authors:** Sayan Ghosal, Michael C. Schatz, Archana Venkataraman

**Affiliations:** 1 Department of Electrical and Computer Engineering, Johns Hopkins University, Baltimore, MD, USA; 2 Department of Computer Science, Johns Hopkins University, Baltimore, MD, USA; 3 Department of Electrical and Computer Engineering, Boston University, Boston, MA, USA

## Abstract

We introduce a novel framework BEATRICE to identify putative causal variants from GWAS summary statistics (https://github.com/sayangsep/Beatrice-Finemapping). Identifying causal variants is challenging due to their sparsity and to highly correlated variants in the nearby regions. To account for these challenges, our approach relies on a hierarchical Bayesian model that imposes a binary concrete prior on the set of causal variants. We derive a variational algorithm for this fine-mapping problem by minimizing the KL divergence between an approximate density and the posterior probability distribution of the causal configurations. Correspondingly, we use a deep neural network as an inference machine to estimate the parameters of our proposal distribution. Our stochastic optimization procedure allows us to simultaneously sample from the space of causal configurations. We use these samples to compute the posterior inclusion probabilities and determine credible sets for each causal variant. We conduct a detailed simulation study to quantify the performance of our framework across different numbers of causal variants and different noise paradigms, as defined by the relative genetic contributions of causal and non-causal variants. Using this simulated data, we perform a comparative analysis against two state-of-the-art baseline methods for fine-mapping. We demonstrate that BEATRICE achieves uniformly better coverage with comparable power and set sizes, and that the performance gain increases with the number of causal variants. Thus, BEATRICE is a valuable tool to identify causal variants from eQTL and GWAS summary statistics across complex diseases and traits.

## Author summary

Fine-mapping provides a way to uncover genetic variants that causally affect some trait of interest. However, correct identification of the causal variants is challenging due to the correlation structure shared across variants. While current fine-mapping approaches take into account this correlation structure, they are often computationally intensive to run and cannot handle spurious effects from non-causal variants. In this paper, we introduce BEATRICE, a novel framework for Bayesian fine-mapping from summary data. Our strategy is to impose a binary concrete prior over the causal configurations that can handle non-zero spurious effects and to infer the posterior probabilities of the causal variant locations using deep variational inference. In a simulation study, we demonstrate that BEATRICE achieves comparable or better performance to the current fine-mapping methods across increasing numbers of causal variants and increasing noise, as determined by the polygenecity of the trait.

## 1 Introduction

Genome-Wide Association Studies (GWAS) provide a natural way to quantify the contribution each genetic variant to the observed phenotype [1]. However, the univariate nature of GWAS does not take into account the correlation structure shared between the genetic variants, which arises from to low genomic recombination of nearby DNA regions [2]. Consequently, strong correlations can inflate the effect size of a non-causal genetic variant, thus leading to false positive identifications [3] Fine-mapping [4, 5] addresses this problem by analyzing the correlation structure of the data to identify small subsets of causal genetic variants [5, 6]. These subsets, known as credible sets, capture the uncertainty of finding the true causal variant within a highly correlated region [7]. Unlike p-values, the corresponding posterior inclusion probabilities (PIPs) computed during fine-mapping can be compared across studies of different sample sizes.

Traditional fine-mapping methods can be grouped into two general categories. The first category uses a penalized regression model to predict the output phenotype based on the collection of genetic variants [8, 9]. Popular regularizations like LASSO [10] and Elastic Net [9] simultaneously perform effect size estimation while slowly shrinking the smaller effect sizes to zero. The drawback of penalized regression models is that they optimize phenotypic prediction and, due to the correlation structure, do not always identify the true causal variants. The second category relies on Bayesian modeling. Here, the phenotype is modeled as a linear combination of the genetic variants, with sparsity incorporated into the prior distribution for the model weights. Approximate inference techniques, such as Markov Chain Monte Carlo (MCMC) [11] and variational methods [12] have been used to infer the effect sizes, PIPs, and credible sets. While these approaches represent valuable contributions to the field, they require the raw genotype and phenotype information, which raises privacy and regulatory concerns, particularly in the cases of publicly shared datasets. MCMC sampling also requires a burn-in period, which adds a substantial (100X) runtime overhead.

In response to these concerns, fine-mapping approaches have moved towards using summary statistics, which can be easily shared across sites. For example, the works of [13–15] use a stochastic or exhaustive search to identify the posterior probabilities of the causal configurations. However, exhaustive search based methods are restricted by the number of assumed causal variants, as this leads to an exponential increase in the dimensionality of the approximate posterior distribution. Stochastic search approaches [13] are computationally less expensive, but, by construction, they cannot handle nontrivial effects from spurious non-causal variants. The most recent contribution to fine-mapping is SuSiE [16, 17], which estimates the variant effect sizes as a sum of “single effects”. These “single effect” vectors contain one non-zero element representing a causal variant and are estimated using a Bayesian step-wise selection approach. SuSiE provides a simple framework to robustly estimate PIPs and credible sets; however, there is limited evidence for its performance given the presence of spurious genetic effects. Such scenarios can appear due to polygenicity of the trait, trans-interactions of variants, or varying correlation structure of the genomic region.

In this paper, we introduce BEATRICE, a novel framework for **B**ayesian fin**E**-mapping from summ**A**ry da**T**a using deep va**R**iational **I**nferen**CE**. In contrast to sampling methods, we approximate the posterior distribution of the causal variants given the GWAS summary statistics as a binary concrete distribution [18, 19], whose parameters are estimated using a deep neural network. This unique formulation allows BEATRICE to use computationally efficient gradient-based optimization to minimize the KL divergence between the proposal binary concrete distribution and the posterior distribution of the causal variants. In addition, our unique optimization strategy samples a representative set of causal configurations in the process of minimizing the empirical KL divergence; these configurations can be used to obtain the PIPs and the credible sets. We compare our model with two state-of-the-art fine-mapping approaches, SuSiE [16] and FINEMAP [13]. We perform an extensive simulation study and quantify the performance of each model across increasing numbers of causal variants and increasing noise, as determined by the degree to which non-causal variants explain the phenotype variance. The runtimes of both SuSiE and BEATRICE are less than one minute, in contrast FINEMAP requires significantly longer to converge. On average BEATRICE achieves 2.2 fold increase in coverage, 0.1 fold increase in AUPRC, and similar power in comparison to SuSiE and FINEMAP.

## 2 Generative Assumptions of Fine-mapping

BEATRICE is based on a generative additive effect model. Formally, let $\text{y}\in {\mathbb{R}}^{n\times 1}$ denote a vector of (scalar) quantitative traits across *n* subjects. The corresponding genotype data $\text{X}\in {\mathbb{R}}^{n\times m}$ is a matrix, where *m* represents the number of genetic variants in the analysis. Without loss of generality, we assume that the the columns of **X** have been normalized to have mean 0 and variance 1, i.e., $\frac{1}{n}\sum_{i}{\text{X}}_{ij}=0$ and $\frac{1}{n}\sum_{i}{\text{X}}_{ij}^{2}=1$ for *j* = 1, …, *m*. The quantitative trait is generated as follows:

(1) $$\text{y}=\text{X}\beta +\eta \;\eta \sim N\left(0,\frac{1}{\tau }{\text{I}}_{n}\right),$$

where $\beta \in {\mathbb{R}}^{m\times 1}$ is the effect size, $\eta \in {\mathbb{R}}^{n\times 1}$ is additive white Gaussian noise with variance *τ*, and **I**_*n*_ is the *n* × *n* identity matrix.

### 2.1 Genome Wide Association Studies (GWAS)

GWAS uses a collection of element-wise linear regression models to estimate the effect of each genetic variant. Mathematically, the GWAS effect sizes are computed as $\hat{\beta }=\frac{1}{n}X^{T}y$, with the corresponding vector of normalized z-scores equal to $Z=\frac{1}{\sqrt{n\tau }}X^{T}y$ [1, 14]. The main drawback of GWAS is that non-causal genetic variants can have large effect sizes due to polygenicity of the quantitative trait [20], varying degrees of linkage disequilibrium (LD) with causal variants [3], and/or interactions of the variant with enriched genes [20]. One popular strategy to mitigate this drawback is to impose a sparse prior over *β* given the set of causal variants:

(2) $$\beta ∼N\left(0,\frac{1}{\tau }\sigma^{2}{\text{Σ}}_{C}\right)$$

(3) $${\text{Σ}}_{C}\left(i,j\right)=\left\{\begin{array}{ll} 0, & i\neq j\\ 1, & i=j\text{ and }i\text{ is causal}\\ \epsilon , & i=j\text{ and }i\text{ is non-causal with non-zero effect}\\ 0, & \text{otherwise} \end{array}\right.$$

Notice from Eq. (3) that the variance of *β*(*i*) for a causal variant is $\frac{\sigma^{2}}{\tau }$ and the variance of *β*(*i*) for a non-causal variant with non-zero effect is $\epsilon \frac{\sigma^{2}}{\tau }$, where ϵ is assumed to be small. This formulation handles residual influences from the non-causal variants, which are often observed in real-world data. Under this assumed prior, we can show [14, 21] that the normalized GWAS effect sizes **z** are distributed as:

(4) $$p\left(\text{z}|{\text{Σ}}_{X},{\text{Σ}}_{C}\right)=N\left(\text{z};0,{\text{Σ}}_{X}+{\text{Σ}}_{X}\left(n\sigma^{2}{\text{Σ}}_{C}\right){\text{Σ}}_{X}\right)$$

where ${\text{Σ}}_{X}=\frac{1}{n}{\text{X}}^{T}\text{X}$ is the empirical correlation matrix of the genotype data, also known as the LD matrix. Broadly, the goal of fine-mapping is to identify the causal variants *i*, i.e., non-zero elements of **Σ**_*C*_ given the effect sizes **z** and the LD matrix **Σ**_*X*_. The derivation is provided in Section S1 of the Supplement.

## 3 Materials and Methods

**Fig 1.**
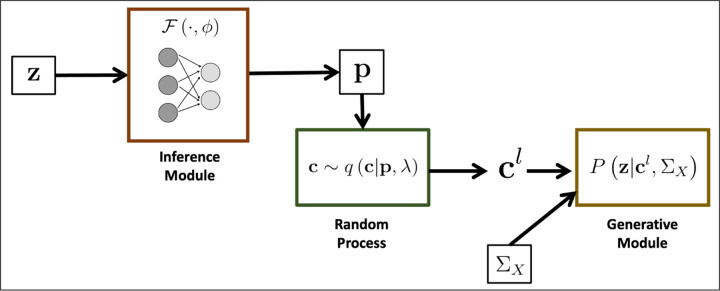
Overview of BEATRICE. The inputs to our framework are the LD matrix **Σ**_*X*_ and the summary statistics **z**. The inference module uses a neural network to estimate the underlying probability map **p**. The random process generates random samples **c**^*l*^ for the Monte Carlo integration in Eq. (12). Finally, the generative module calculates the likelihood of the summary statistics from the sample causal vectors **c**^*l*^.

BEATRICE uses a variational inference framework for fine-mapping. For convenience, we represent the diagonal elements of **Σ**_*C*_ by the vector $\text{c}\in {\mathbb{R}}^{m\times 1}$, and by construction, **c** encodes the causal variant locations. Figure 1 provides an overview of BEATRICE. Our framework consists of three main components: an inference module, a random sampler, and a generative module. The inputs to BEATRICE are the summary statistics **z** and the LD matrix **Σ**_*X*_. The inference module estimates the parameters **p** of our proposal distribution *q* (·; **p**, *λ*) using a neural network. The random process sampler uses the parameters **p** to randomly sample potential causal vectors **c** according to the given proposal distribution. Finally, the generative module calculates the likelihood of the observed summary statistics **z** according to Eq. (4).

### 3.1 Proposal Distribution

The goal of fine-mapping is to infer the posterior distribution *p*(**c**|{**z**, **Σ**_**X**_}), where **c** corresponds to the diagonal elements of **Σ**_*C*_. Due to the prior formulation in Eqs. (2–3), solving for the true posterior distribution is computationally intractable, as it requires a combinatorial search over the possible causal configurations. Thus, we approximate the posterior distribution *p*(**c**|{**z**, **Σ**_**X**_}) with a binary concrete distribution *q*(**c**; **p**, *λ*) [18], where the parameters **p** of the distribution are functions of the inputs {**z**, **Σ**_**X**_}. Samples **c** generated under a binary concrete distribution can be viewed as continuous relaxations of independent Bernoulli random variables. This reparametrization [19] allows us to learn **p** from the data using standard gradient descent.

Formally, let **c**_*i*_ and **p**_*i*_ denote the *i*th element of the vectors **c** and **p**, respectively. Each entry of **c** is independent and is distributed as follows:

(5) $$q\left(c_{i};p_{i},\lambda \right)=\frac{\lambda p_{i}c_{i}^{-\lambda -1}\left(1-p_{i}\right){\left(1-c_{i}\right)}^{-\lambda -1}}{{\left(p_{i}c_{i}^{-\lambda }+\left(1-p_{i}\right){\left(1-c\right)}^{-\lambda }\right)}^{2}},$$

where the parameter *λ* controls the extent of relaxation from a Bernoulli distribution.

We can easily sample from the binary concrete distribution in Eq. (5) via

(6) $$c_{i}=\xi \left(\frac{\log\left(\frac{U}{1-U}\right)+\log\left(\frac{p_{i}}{1-p_{i}}\right)}{\lambda }\right),$$

where *ξ*(·) is the sigmoid function, and the random variable *U* is sampled from a uniform distribution over the interval [0, 1]. As seen, **p**_*i*_ specifies the underlying probability map and *U* provides stochasticity for the sampling procedure in Eq. (6). We note that the gradient of Eq. (6) with respect to **p**_*i*_ tends to have a low variance in practice, which helps to stabilize the optimization.

The two unique properties of binary concrete random variable are $P\left({\text{c}}_{i}>\frac{1}{2}\right)={\text{p}}_{i}$ and $\lim_{\lambda \to 0}P\left({\text{c}}_{i}=1\right)={\text{p}}_{i}$. The first property indicates that **p**_*i*_ controls the degree to which **c**_*i*_ assumes low values close to 0 and high values close to 1. This property also give BEATRICE flexibility to handle genetic variants with different levels of association, thus aligning with our generative process that assumes some non-causal variants may have small non-zero effects. The second property implies that a high probability **p**_*i*_ at location *i* is highly indicative of a causal variant. Taken together, the binary concrete distribution has an easily-optimized parameterization with desirable properties.

### 3.2 Variational Inference

We select the variational parameters {**p**, *λ*} to minimize the Kullback–Leibler (KL) divergence between the proposal distribution and the posterior distribution of the causal vector **c** given the input data {**z**, **Σ**_**X**_}, that is

(7) $$\left\{{\text{p}}^{*},\lambda^{*}\right\}=\arg\underset{\left\{\text{p},\lambda \right\}}{\min}KL\left(q\left(\text{c};\text{p},\lambda \right)||p\left(\text{c}|\left\{\text{z},{\text{Σ}}_{\text{X}}\right\}\right)\right)$$

Using Bayes’ Rule, we can show that the optimization in Eq. (7) can be rewritten

(8) $$\left\{{\text{p}}^{*},\lambda^{*}\right\}=\arg\underset{\left\{\text{p},\lambda \right\}}{\min}KL\left.\left(q\left(\text{c};\text{p},\lambda \right)||p\left(\text{c};{\text{p}}_{0},\lambda_{0}\right)\right)\right)-E_{q\left(\cdot ;\text{p},\lambda \right)}\left[\log\left(p\left(\text{z}|{\text{Σ}}_{\text{X}},\text{c}\right)\right)\right],$$

where we have assumed an element-wise binary concrete prior *p*(**c**; **p**_0_, *λ*_0_) over the vector **c**. We fix the relaxation parameter to be small (*λ* = 0.01) and the probability map to be uniform ${\text{p}}_{0}={\left[\frac{1}{m},\ldots ,\frac{1}{m}\right]}^{\text{T}}$. Thus, the first term of Eq. (8) can be viewed as a regularizer that encourages sparsity in causal vectors **c**. The second term of Eq. (8) can be interpreted as the likelihood of the observed test statistics. The works of [22, 23] have demonstrated that under certain assumptions, the likelihood term of the summary statistics is the same as the original data likelihood *p* (**y|X**, **c**) derived from Eq. (1).

During optimization, the relaxation parameter *λ* is annealed [18, 19] to a small non-zero value (0.01) with fixed constant rate, and the underlying probability map **p** is optimized using gradient descent. Specifically, we use a neural network to generate the vector $\text{p}=ℱ\left(\text{z};\phi \right)$. The details of the neural network architechture are provided in Section S3 of the Supplement. Optimizing p* now amounts to optimizing the learnable parameters of the neural network *ϕ*. Given a fixed value of *λ*, the neural network loss function follows from Eq. (8) according to

(9) $$\left.ℒ(\phi )=KL\left(q(c;p(\phi ),\lambda )∥p\left(c;p_{0},\lambda_{0}\right)\right)\right)-E_{q(\cdot ;p(\phi ),\lambda )}\left[\log\left(p\left(z|\Sigma_{X},c\right)\right)\right],$$

where we have defined p(ϕ)≜ℱ(z;ϕ) for notational convenience.

### 3.3 Optimization Strategy

The expectations in Eq. (9) do not have closed-form expressions. Therefore, we use Monte Carlo integration to accurately approximate $ℒ\left(\phi \right)$ in the regime of small *λ*, i.e., when the binary concrete distribution behaves similar to a Bernoulli distribution.

Let **c**^1^(*ϕ*), …, **c**^*L*^(*ϕ*) be a collection of causal vectors sampled independently from *q*(·|**p**(*ϕ*), *λ*) according to Eq. (6). The likelihood term of Eq. (9) is computed as

(10) $$E_{q\left(\cdot ;\text{p}\left(\phi \right),\lambda \right)}\left[\log\left(p\left(\text{z}|{\text{Σ}}_{\text{X}},\text{c}\right)\right)\right]=\frac{1}{L}\sum_{l=1}^{L}\log\left(p\left(\text{z}|{\text{Σ}}_{\text{X}},{\text{c}}^{l}\left(\phi \right)\right)\right),$$

where the right-hand side probability is computed according to Eq. (4) by substituting **c**^*l*^(*ϕ*) for the diagonal entries of **Σ**_*C*_ in each term of the summation. Once again, the continuous relaxation used to generate **c**^*l*^(*ϕ*) in Eq. (6) allows us to directly optimize *ϕ*.

We approximate the first term of Eq. (9) under the assumption of small {*λ*, *λ*_0_} on the order of 0.01. In this case, the binary concrete distribution behaves like a 0, 1 Bernoulli distribution. Under these conditions, we can write the first term of Eq. (9) as

(11) $$\begin{array}{l} KL\left.\left(q\left(\text{c};\text{p}\left(\phi \right),\lambda \right)||p\left(\text{c};{\text{p}}_{0},\lambda_{0}\right)\right)\right)\\ \approx \sum_{i=1}^{m}\left[{\text{p}}_{i}\left(\phi \right)\log\left(\frac{{\text{p}}_{i}\left(\phi \right)}{p_{0}}\right)+\left(1-{\text{p}}_{i}\left(\phi \right)\right)\log\left(\frac{1-{\text{p}}_{i}\left(\phi \right)}{1-p_{0}}\right)\right], \end{array}$$

where *p*_0_ is a fixed scalar parameter used to construct the (constant) prior vector **p**_0_. We note that the criteria {*λ* → 0.01, *λ*_0_ = 0.01} is satisfied in practice, as *λ* is annealed during the optimization to progressively smaller values and *λ*_0_ is fixed *a priori*.

The above approximations allow us to rewrite the neural network loss as

(12) $$\begin{array}{l} ℒ(\phi )\approx -\frac{1}{L}\sum_{l=1}^{L}\log N\left(\text{z};0,{\text{Σ}}_{X}+{\text{Σ}}_{X}\left(n\sigma^{2}{\text{Σ}}_{C}^{l}(\phi )\right){\text{Σ}}_{X}\right)\\ \;+\sum_{i=1}^{m}p_{i}(\phi )\log\left(\frac{p_{i}(\phi )}{p_{0}}\right)+\left(1-p_{i}(\phi )\right)\log\left(\frac{1-p_{i}(\phi )}{1-p_{0}}\right), \end{array}$$

where ${\text{Σ}}_{C}^{l}\left(\phi \right)$ corresponds to the diagonal matrix using the vector **c**^*l*^(*ϕ*) as the diagonal entries. We use a stochastic gradient descent optimizer [24] to minimize the loss $ℒ\left(\phi \right)$ with respect to the neural network weights *ϕ*. This process is detailed in Algorithm 1.


$$\bar{\underline{\begin{array}{l} \mathrm{Algorithm 1}\text{ Optimization scheme to minimize Eq. }\left(12\right)\\ \;{ℬ}^{R}=\left\{\;\right\}\\ \text{ Initialize }\phi_{0}\\ \text{ for }t=\left[1\ldots T\right]\text{ do}\\ \text{ Generate }p\left(\phi_{t}\right)=ℱ\left(\text{z};\phi_{t}\right)\\ \text{ Randomly sample }{\text{c}}_{t}^{l}\text{ according to Eq}.\;\left(6\right)\\ \text{ Binarize }{\text{c}}_{t}^{l}\text{ to }{\text{b}}_{t}^{l}\text{ and add to }{ℬ}^{R}\\ \;{𝒮}_{t}^{l}=\left\{i\right\}\;s.t.\;{\text{c}}_{t}^{l}\left(i\right)>0.01\\ \text{ Prune set }{𝒮}_{t}^{l}\text{ such that it consists of 50 indices.}\\ \;{\text{c}}_{t}^{l}\left(i\right)=0\text{ if i}\notin {𝒮}_{t}^{l}\\ \text{ Generate }ℒ\left(\phi_{t}\right)\text{ according to Eq. }\left(12\right)\\ \;\phi_{t+1}=\phi_{t}-StepSize\nabla ℒ\left(\phi^{t}\right)\\ \text{ end for} \end{array}}}$$

### 3.4 Computational Complexity

Each iteration of stochastic gradient descent requires us to compute the likelihood term $\left[\log N\left(\text{z};0,{\text{Σ}}_{X}+{\text{Σ}}_{X}\left(n\sigma^{2}{\text{Σ}}_{C}^{l}\left(\phi \right)\right){\text{Σ}}_{X}\right)\right]$. This computation is expensive due to the covariance matrix inversion, whose run-time is on the order of *O*(*m*^3^), where *m* is the total number of variants. To mitigate this issue, the works of [25] show that if ${\text{Σ}}_{C}^{l}\left(\phi \right)$ is sparse, then the matrix inversion can be done with order *O*(*k*^3^) + *O*(*mk*^2^) run-time, where *k* is the number of non-zero diagonal elements of ${\text{Σ}}_{C}^{l}\left(\phi \right)$. We leverage this result in the optimization by thresholding the elements of **c**^*l*^(*ϕ*) to set small values exactly to zero. We specify this threshold such that at most 50 elements of **c**^*l*^(*ϕ*) are non-zero at each iteration. This choice allows us to run BEATRICE in fixed time for all scenarios for a fixed *m*. We also regularize **Σ**_*X*_ with a small diagonal load to ensure invertibility of the covarance matrix at each iteration. Finally, we run stochastic gradient descent with a batch size of one to further speed up BEATRICE. Effectively, this means that we sample a single **c**^*l*^(*ϕ*) at each epoch rather than perform a true Monte Carlo integration. The authors of [26] have previously shown that a single random sample (*L* = 1) is sufficient to guarantee convergence to a local minimum of Eq. (12). Algorithm 1 provides a detailed description of these optimization steps.

### 3.5 Causal Configurations and Posterior Inclusion Probabilities

The desired outputs of each fine-mapping method are Posterior Inclusion Probabilities (PIPs) and credible sets. PIPs estimate how likely each variant is causal as a measure of its importance. Credible sets identify the subset of variants that are likely to contain a causal variant, which captures the uncertainty of finding the true variant.

The main challenge to estimating the posterior probability of a given causal configuration (i.e., set of causal variant locations) is the exponentially large search space. Let **b** denote a binary vector with a value of 1 at causal locations and a value of 0 at non-causal locations. At a high level, **b** can be viewed as a binarized version of the causal vector **c** in the previous sections. Using Bayes’ Rule, the posterior probability of **b** given the input data {**z**, **Σ**_**X**_} can be written as follows:

(13) $$p\left(b|z,\Sigma_{X}\right)=\frac{p\left(z|\Sigma_{X},b\right)p(b)}{\sum_{{\text{b}}^{\prime }\in ℬ}p\left(z|\Sigma_{X},b^{\prime }\right)p\left(b^{\prime }\right)}$$

where ℬ is the set of all 2^*m*^ possible causal configurations. Once again, **z** captures the summary statistics and **Σ**_*X*_ is the LD matrix. Even though ℬ is exponentially large, it has been argued [27] that the majority of these configurations have negligible probability and do not contribute to the denominator of Eq. (13).

Our stochastic optimization provides a natural means to track causal configurations with non-negligible probability to compute *p* (**b**|**z**, **Σ**_*X*_). Namely, at each iteration of stochastic gradient descent, we randomly generate a sample causal vector **c**^*l*^ to minimize Eq. (12). In parallel, we binarize the vector **c**^*l*^ via

$${\text{b}}_{i}^{l}=\left\{\begin{array}{ll} 1, & {\text{c}}_{i}^{l}>\gamma ,\\ 0, & otherwise \end{array}\right.$$

and add the resulting vector **b**^*l*^ to a reduced set of causal configurations ${ℬ}^{R}$. The variational objective ensures that our proposal distribution converges to the true posterior distribution of the causal vectors. Thus, the samples **c**^*l*^ lie near modes of the posterior distribution which is the neighborhood of non-negligible probability.

In this work, we use a threshold *γ* = 0.1 to binarize the vectors **c**^*l*^. Empirically, we find that this threshold value preserves the main interactions between variants. However, the user of BEATRICE can adjust this threshold as needed.

After obtaining the sampled vectors, we replace the exhaustive set **B** in Eq. (13) with the reduced set ${ℬ}^{R}$ for tractable computation of *p* (**b**|**z**, **Σ**_*X*_). We then compute the posterior inclusion probability (PIP) of each variant by summing the probabilities over the subset of ${ℬ}^{R}$ with a value of 1 at that variant location. Mathematically,

(14) $$P\left(b_{i}=1|z,{\text{Σ}}_{X}\right)\approx \sum_{\text{b}\in 𝒮}p\left(b|z,\Sigma_{X}\right)$$

(15) $$\text{s.t. }𝒮\subset {ℬ}^{R}\text{ and }𝒮=\left\{b|b_{i}=1\right\}$$

where 𝒮 is a subset of ${ℬ}^{R}$ that contains binary configurations with 1 at location *i*.

Finally, we identify the credible sets in two steps. First, in a conditional step-wise fashion, we identify the variants with the highest conditional probability given the previously selected variants. This strategy identifies the set of “key” variants with a high probability of being causal. Second, we determine the credible set for each key variant, by computing the conditional inclusion probabilities of each variant given the key variants and adding variants to the credible set. A detailed description of this process can be found in the Supplementary Methods document (Section S2 **in** S1 text).

### 3.6 Baselines

We compare our approach with the state-of-the-art methods, FINEMAP and SuSiE.

#### FINEMAP:

This approach uses a stochastic shotgun search to identify causal configurations with non-negligible posterior probability. FINEMAP defines the neighborhood of a configuration at every step by deleting, changing or adding a causal variant from the current configuration. The next iteration samples from this neighborhood, thus reducing the exponential search space to a smaller high-probability region. Finally, the identified causal configurations are used to determine the posterior inclusion probabilities for each variant. The computationally efficient shotgun approach makes FINEMAP a viable tool for finemapping from multiple GWAS summary data in [28, 29]. However, the FINEMAP algorithm [13] does not provide definitive credible sets, thus we rely on the same approach used in [16] for this task. Details of this procedure are provided in Section S4 of the Supplement.

#### SuSiE:

The recent works of [16, 17] introduced an iterative Bayesian selection approach for fine-mapping that represents the variant effect sizes as a sum of “single-effect” vectors. Each vector contains only one non-zero element, which represents the causal signal. In addition to finding causal variants, SuSiE provides a way to quantify the uncertainty of the causal variants locations via credible sets. SuSiE has also been used widely to find putative causal variants GWAS summary statistics [30, 31].

### 3.7 Evaluation Strategy

We evaluate several metrics of performance in our simulation study.

#### Area Under Precision Recall Curve (AUPRC):

We threshold the PIPs and compute the precision and recall for identifying the ground-truth causal configuration. High precision indicates a low false positive rate, while high recall relates to a low false negative rate. Thus, the AUPRC, which is computed by sweeping the PIP threshold, can be viewed as a holistic measure of performance across both classes. AUPRC is also robust to severe class imbalance [32], which is the case in fine-mapping.

#### Coverage, Power and Size of the Credible Sets:

We assess the quality of the credible sets using three metrics [16, 17]. Coverage is the percentage of credible sets that contain at least one true causal variant, and power is the percentage of ground-truth causal variants that appear in at least one credible set. Higher coverage indicates that the method is more confident about its prediction of causal variants, whereas higher power indicates the method can accurately identify all the causal variants.

One caveat is that we can generally achieve both higher coverage and higher power simply by adding variants to the credible sets. To counter this trend, we report the average size of the credible sets identified by each method. Ideally, we would like the credible sets to be as small as possible while retaining high coverage and high power.

## 4 Experimental Setup

### Genotype Simulations:

We use the method of [33] to simulate genotypes **X** based on data from the 1000 Genomes Project. We select an arbitrary sub-region (39.9*Mb* − 40.9*Mb*) from Chromosome 2 as the base. After filtering for rare variants (MAF *<* 0.02), the remaining 3.5K variants are used to simulate pairs of haplotypes to generate 10,000 unrelated individuals. In each experiment below, we randomly select *m* = 1*k* variants and *n* = 5000 individuals to generate the phenotype data.

### Phenotype Generation:

We generate the phenotype **y** from a standard mixed linear model [22], where the influences of the causal variants are modeled as fixed effects, and the influences of other non-causal variants are modeled as random effects. In this case, the genetic risk for a trait is spread over the entire dataset, with each variant having small individual effects, as per the polygenicity assumption of a complex trait.

Given a set of *d* causal variants *C*, let **X**_*C*_ ∈ **R**^*n*×*d*^ denote the corresponding subset of the genotype data and **X**_*NC*_ ∈ **R**^*n*×*m*−*d*^ denote the remaining non-causal variants. From here, we generate the phenotype data **y** as follows:

$$\text{y}={\text{X}}_{C}\beta +{\text{g}}_{NC}+\epsilon ≜{\text{g}}_{C}+{\text{g}}_{NC}+\epsilon$$

$$g_{NC}∼N\left(0,\frac{1}{m-d}X_{NC}X_{NC}^{T}\right)$$

$$\beta ∼N\left(0,I_{d}\right)$$

$$\epsilon ∼N\left(0,\alpha^{2}I_{n}\right)$$

where *β* is the *d*-dimensional effect sizes sampled from a Gaussian, and *ϵ* is an zero-mean Gaussian noise with variance *α*^2^. The random variable **g**_*NC*_ models the effect of the non-causal variants as a multivariate Gaussian vector with mean 0 and covariance $\frac{1}{m-d}{\text{X}}_{NC}{\text{X}}_{NC}^{T}$. Likewise, **g**_*C*_ = **X**_*C*_ *β* captures the effect of the causal variants.

In our experiments, we define *ω*^2^ as the total phenotypic variance attributed to the genotype (e.g., both **g**_*C*_ and **g**_*NC*_) and *p* as the proportion of this variance associated with the causal variants in **g**_*C*_. Using the strategy described in [34], we enforce these conditions by normalizing the phenotype **y** as follows:

(16) $$\tilde{y}=\sqrt{\frac{p\omega^{2}}{var\left(g_{C}\right)}}g_{C}+\sqrt{\frac{(1-p)\omega^{2}}{var\left(g_{NC}\right)}}g_{NC}+\tilde{\epsilon }$$

$$\tilde{\epsilon }∼N\left(0,\left(1-\omega^{2}\right){\text{1}}_{n}\right)$$

where *var*(**g**_*C*_) and *var*(**g**_*NC*_) are the empirical variances of **g**_*C*_ and **g**_*NC*_, respectively.

After generating the genotype **X** and the normalized phenotype $\tilde{\text{y}}$, we run a GWAS to estimate the effect size ${\hat{\beta }}_{i}$ of each variant *i*. From here, we convert the estimated effect sizes to z-scores via ${\text{z}}_{i}=\frac{{\hat{\beta }}_{i}}{se\left({\hat{\beta }}_{i}\right)}$, where *se*(·) denotes the standard error. The LD matrix is computed from the genotype data as ${\text{Σ}}_{X}=\frac{1}{n}{\text{X}}^{T}\text{X}$. The z-scores and LD matrix are input to each of the fine-mapping methods above.

### Noise Configurations:

We evaluate the performance of each method while varying the number of causal variants *d*, the total genotype variance *ω*^2^, and the proportion of this variance associated with the causal variants *p*. Formally, we sweep over one order of magnitude for *d* = [1, 4, 8, 12], *ω*^2^ = [0.1, 0.2, 0.4, 0.5, 0.7, 0.8], and *p* = [0.1, 0.3, 0.5, 0.7, 0.9]. For each noise setting, we randomly generate 20 datasets by independently re-sampling the causal variant locations, the effect sizes {*β*_*i*_}, the non-causal component **g**_*NC*_, and the noise $\tilde{\epsilon }$. We run all three fine-mapping methods over a total of 4 × 6 × 5 × 20 = 2400 configurations for a comphrehensive evaluation.

## 5 Results

### Varying the Number of Causal Variants

**Fig 2.**
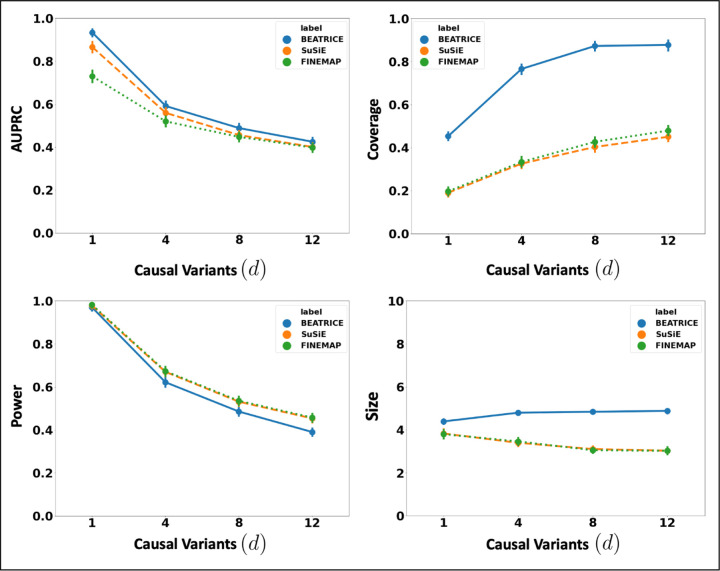
The performance metrics for the three methods across varying numbers of causal variants. Along the x-axis, we plot the number of causal variants, and across the y-axis, we plot the mean and confidence interval (95%) of each metric. We calculate the mean by fixing *d* to a specific value *d* = d* and sweep over all the noise settings where *d* = d*.

Figure 2 illustrates the performance of each method (BEATRICE, FINEMAP, and SuSiE) while increasing the number of causal variants from *d* = 1 to *d* = 12. The points denote the mean performance across all noise configurations (*ω*^2^, *p*) for fixed *d*, and the error bars represent the 95% confidence interval across these configurations. We note that BEATRICE achieves a uniformly higher AUPRC than both baseline method, which suggests that BEATRICE can better estimate the PIPs than FINEMAP or SuSiE. BEATRICE also provides 0.9 – 1.4 fold increase in coverage than the baselines with similar power, which indicates that the credible sets generated by BEATRICE are more likely to contain a causal variant as compared to SuSiE and FINEMAP. Finally, we note that although FINEMAP and SuSiE identify smaller credible sets, the difference in set size between them and BEATRICE is *<* 2 variants. Taken together, as the number of causal variants increases, BEATRICE gives us a better estimate of the PIPs and arguably better credible sets. Compared to the baselines BEATRICE does not impose any prior assumptions over the total number of causal variants, which may lead to its improved performance.

### Increasing the Genotype Contribution:

**Fig 3.**
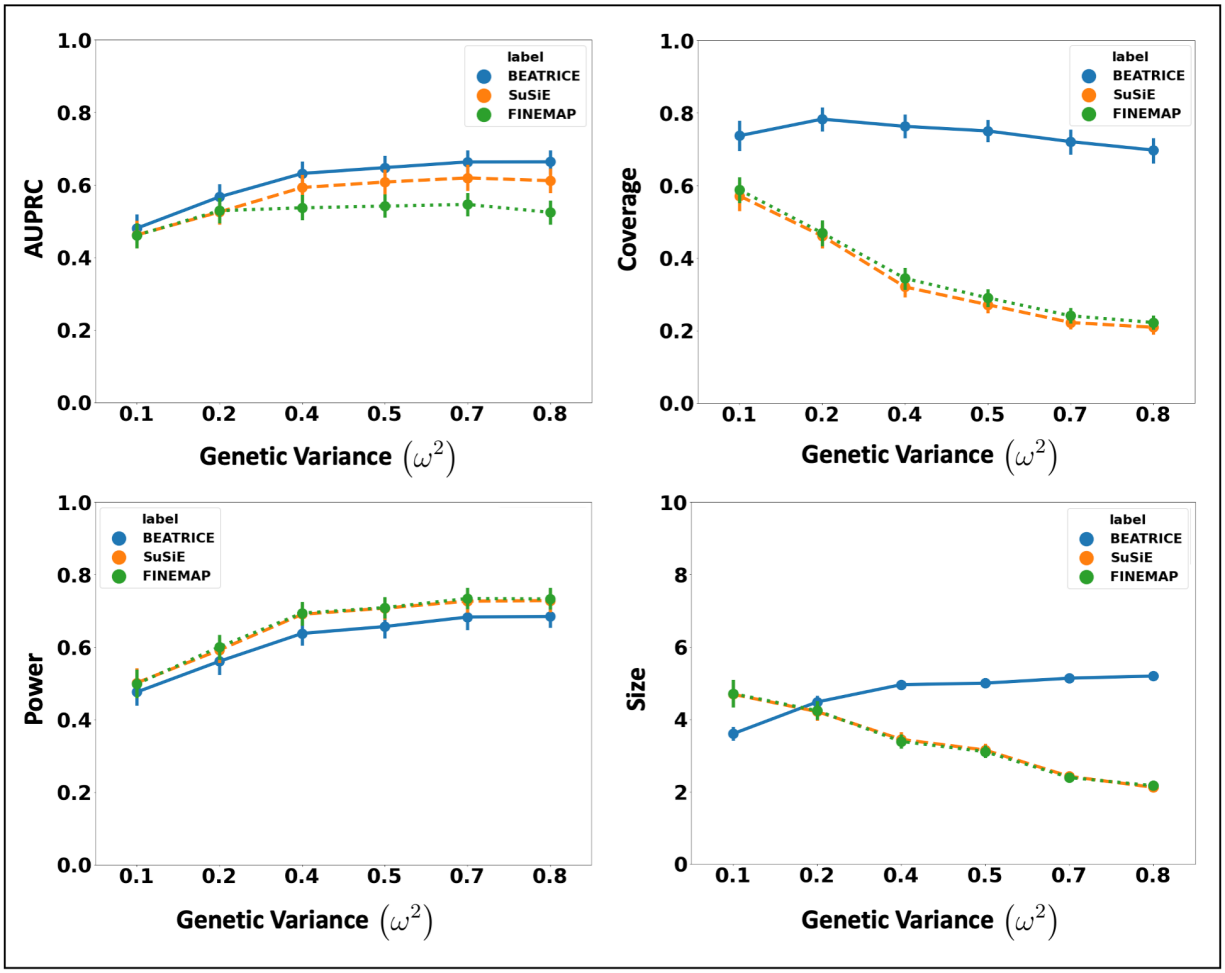
The performance metric for increasing phenotype variance explained by genetics. Along the x-axis, we plot the variance explained by genetics (*ω*^2^), and across the y-axis, we plot each metric’s mean and confidence interval (95%). We calculate the mean by fixing *ω*^2^ to a specific value *ω* = ω* and sweep over all the noise settings where *ω* = ω*.

Figure 3 shows the performance of each method while increasing the genetically-explained variance from *ω*^2^ = 0.1 to *ω*^2^ = 0.8. Similar to above, the points denote the mean performance across all configurations (*d*, *p*) for fixed *ω*^2^, and the error bars represent the 95% confidence interval across these configurations. We note that BEATRICE achieves a significantly higher AUPRC than FINEMAP and a slightly higher AUPRC than SuSiE. When evaluating the credible sets, we observe similar trends in coverage (BEATRICE is 0.25 – 2.34 folds higher) and power (similar performance across methods). While the FINEMAP and SuSiE identify slightly smaller credible, the difference to BEATRICE is only a few variants. Taken together, we submit that BEATRICE achieves the best trade-off across the four performance metrics.

### Varying the Contributions of Causal and Non-Causal Variants:

**Fig 4.**
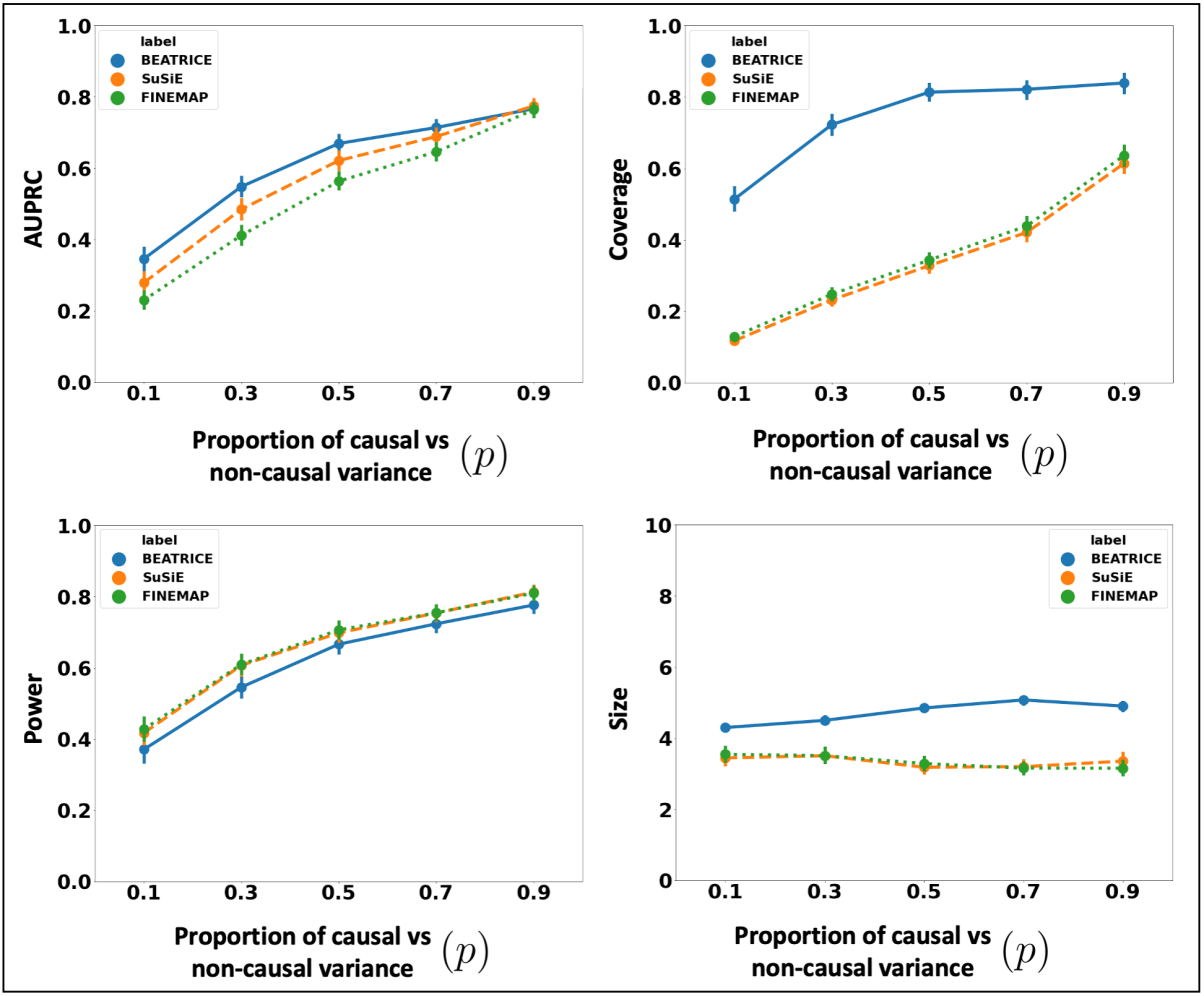
The performance metric for multiple levels of noise introduced by non-causal variants. The noise level (*p*) is explained by the variance ratio of non-causal variants vs. causal variants. Along the x-axis, we plot the noise level (*p*); across the y-axis, we plot each metric’s mean and confidence interval (95%). We calculate the mean by fixing *p* to a specific value *p* = p* and sweep over all the noise settings where *p* = p*.

Figure 4 illustrates the performance of each method while increasing the contribution of the causal variants from *p* = 0.1 to *p* = 0.9. Once again, the points denote the mean performance across all configurations configurations (*d*, *ω*^2^) for fixed *p*, and the error bars represent the 95% confidence interval across these configurations. From an application standpoint, the presence of non-causal variants with small non-zero effects makes it difficult to detect the true causal variants. Accordingly, we observe a performance boost across all methods when *p* is larger. Similar to our previous experiments, BEATRICE provides the best AUPRC, with converging performance as *p* → 1. In addition, BEATRICE identifies better credible sets with significantly higher coverage while maintaining power. Thus, we conclude that BEATRICE is the most robust of the three methods to the presence of noise from non-causal variants. This performance gain may arise from our binary concrete proposal distribution for the causal vector **c**, which provides flexibility to accommodate varying degrees of association.

## 6 Discussion

BEATRICE is a novel, robust, and general purpose tool for fine-mapping that can be used across a variety of studies. One key contribution of BEATRICE over methods like FINEMAP and SuSiE is its ability to discern spurious effects from non-causal variants, including non-causal variants in high LD with true causal variants. Our simulated experiments capture this improved performance by sweeping the proportion of the observed variance attributed to causal (fixed effects) and non-causal (random effects) genetic variants. This parameter *p* ∈ [0, 1] is swept over its natural domain, such that *p* = 1 implies that the only link between the genotype and phenotype comes from the causal variants. At this extreme, Figure 4 shows that all methods achieve comparable performance. However, as *p* decreases, meaning that the effects of non-causal variants increase, BEATRICE outperforms both baselines.

**Fig 5.**
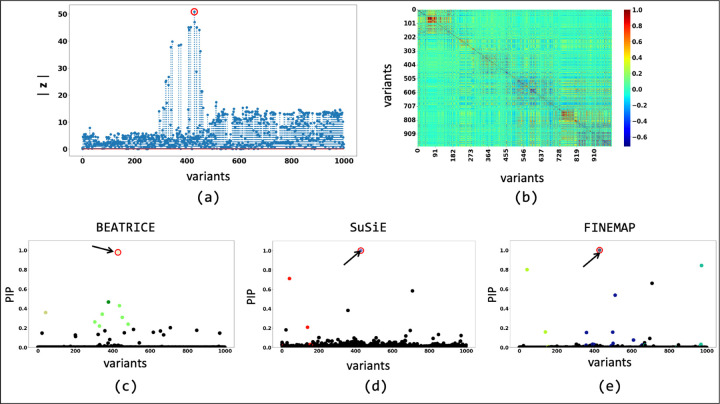
The fine-mapping performance of BEATRICE, SuSiE, and FINEMAP at a noise setting of {*d* = 1, *ω*^2^ = 0.2, *p* = 0.9}. (a) The absolute z-score of each variant as obtained from GWAS. (b) Pairwise correlation between the variants. (c), (d), and (e) are the posterior inclusion probabilities of each variant as identified by BEATRICE, SuSiE, and FINEMAP, respectively. The red circle marked by an arrow shows the location of the causal variant. We have further color-coded the variants based on their assignment to credible sets. The non-black markers represent the variants assigned to a credible set. Additionally, the variants in a credible set are marked by the same color.

**Fig 6.**
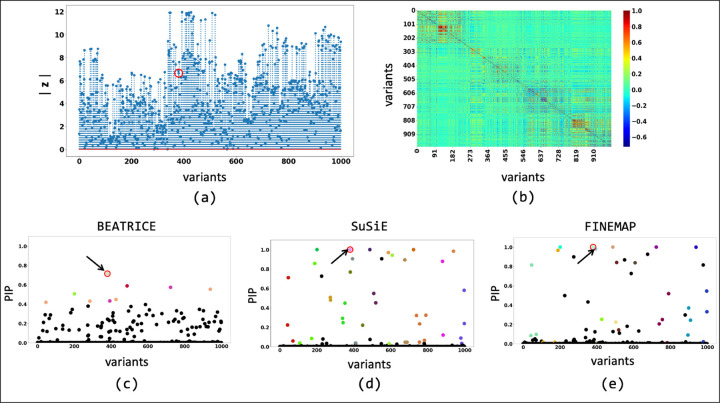
The fine-mapping performance of BEATRICE, SuSiE, and FINEMAP at a noise setting of {*d* = 1, *ω*^2^ = 0.2, *p* = 0.1}. (a) The absolute z-score of each variant as obtained from GWAS. (b) Pairwise correlation between the variants. (c), (d), and (e) are the posterior inclusion probabilities of each variant as identified by BEATRICE, SuSiE, and FINEMAP, respectively. The red circle marked by an arrow shows the location of the causal variant. We have further color-coded the variants based on their assignment to credible sets. The non-black markers represent the variants assigned to a credible set. Additionally, the variants in a credible set are marked by the same color.

We further probe this behavior by illustrating the element-wise PIPs and the credible sets identified by all three methods under two simulation settings: {*d* = 1, *ω*^2^ = 0.2, *p* = 0.9} (Figure 5) and {*d* = 1, *ω*^2^ = 0.2, *p* = 0.1} (Figure 6). As seen in Figure 5, the variance explained by the non-causal variants is small, so the causal variant is easy to distinguish and has been correctly identified by all three approaches. In contrast, we see in Figure 6 that when the non-causal variants play a larger role, the causal variant no longer has the maximum GWAS *z*-score. Here, only BEATRICE correctly identifies the causal variant and assigns it the highest PIP. Both FINEMAP and SuSiE give uncertain predictions, as captured by the large credible sets and multiple high PIPs. We conjecture that BEATRICE takes advantage of the binary concrete distribution to model non-causal variants with non-zero effects, while using the sparsity term of $ℒ\left(\cdot \right)$ to prioritize potentially causal variants.

A second contribution of BEATRICE is our strategic integration of neural networks within a larger statistical framework. Specifically, we use the neural network in Figure 1 as an inference engine to estimate the parameters **p** of our proposal distribution. In this case, the standard over-parameterization in the neural network helps BEATRICE to manage the complexity of the data while providing a buffer against overfitting. BEATRICE leverage the continuous representation of the causal vectors **c**^*l*^ to backpropagate the gradients through the random sampler and train the network. Additionally, the continuous representation of **c**^*l*^ results in low-variance gradients with respect to the underlying probability map, thus leading to a stable optimization.

**Fig 7.**
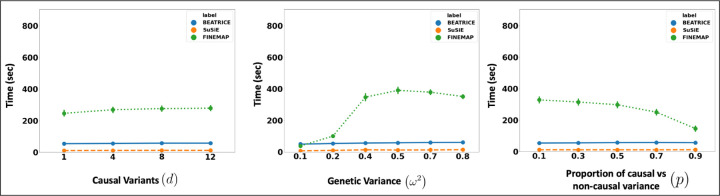
The runtime comparison of BEATRICE, SuSiE, and FINEMAP across all the simulation settings.

Related to the above point, a third contribution of BEATRICE is its ability to efficiently build and evaluate a representative set of causal configurations during the optimization process. This set identifies key regions of the exponential search space to compute the PIPs and credible sets. In particular, we keep track of the sampled vectors at every iteration of the optimization, as described in Section 3.5. By minimizing the KL divergence between the proposal distribution and the true posterior distribution, we ensure that the randomly sampled causal vectors slowly converge to the causal configurations that have non-negligible posterior probability. Our strategy lies is in stark contrast with traditional mean-field approaches, where independence assumptions between elements of the proposal distribution do not allow for joint inference of the causal configurations. Furthermore, this strategy allows us to efficiently estimate the PIPs in finite run-time. Figure 7 compares the average run-time of each method across all parameter settings. We observe that the run-time of BEATRICE and SuSiE are less than one minute. In contrast, FINEMAP requires significantly more time to converge.

The final contribution of BEATRICE is its simple and flexible design. Importantly, BEATRICE can easily incorporate priors based on the functional annotations of the variants. Formally, in the current setup, the prior over **c** is effectively constant, as captured by $p_{0}=\frac{1}{m}$. We can integrate prior knowledge simply by modifying the distribution of *p*_0_ across the variants. Thus, BEATRICE is a general-purpose tool for fine-mapping. Going one step further, a recent direction in fine-mapping is to aggregate data across multiple studies to identify causal variants [25]. Here, different LD matrices across studies helps to refine the fine-mapping results. BEATRICE can be applied in this context as well simply by modifying Eq. (12) as follows:

(17) $$ℒ(\phi )=-\frac{1}{SL}\sum_{s=1}^{S}\sum_{l=1}^{L}\log\left(N\left(z_{s};0,\Sigma_{X_{s}}+\Sigma_{X_{s}}\left(n\sigma^{2}\Sigma_{C}^{l}(\phi )\right)\Sigma_{X_{s}}\right)\right)\;\;+\sum_{i}p_{i}\log\left(\frac{p_{i}}{p_{0}}\right)+\left(1-p_{i}\right)\log\left(\frac{1-p_{i}}{1-p_{0}}\right)$$

where *s* denotes each separate study, *S* is the total number of studies in the analysis, and **z**_*s*_, ${\text{Σ}}_{X_{s}}$ are the summary statistics for each study.

## 7 Code Availability

**Fig 8.**
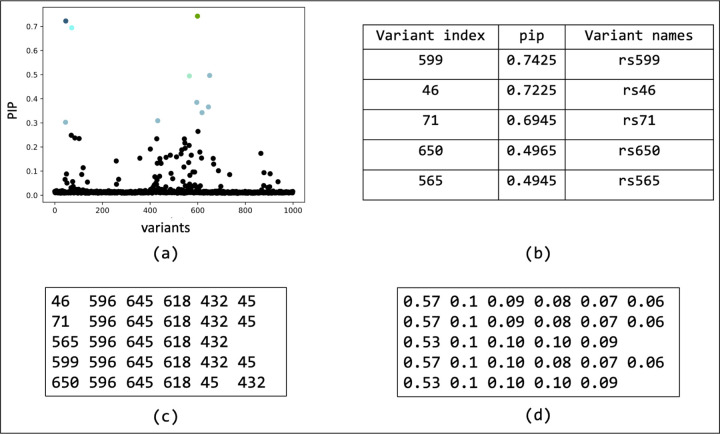
Overview of the outputs generated by BEATRICE. (a) The PIPs are displayed and color coded by their assignment to credible sets. (b) A table with the PIPs and the corresponding name of the variants. (c) A text file with the credible sets. Here each row represent a credible set and the entries are indices of the variants present in the credible set. The first column of each row represents the key index. (d) The conditional inclusion probability of each of the credible variants given the key variant. The calculations can be found in Section S2 of the Supplements.

We have compiled the code for BEATRICE and its dependencies into a docker image, which can be found at https://github.com/sayangsep/Beatrice-Finemapping. We have also provided installation instructions and a detailed description of the usage. The compact packaging will allow any user to directly download and run BEATRICE on their data. Namely, all the user must specify are a directory path to the summary statistics (i.e., z-scores), the LD matrix, and the number of subjects. Fig. 8 shows the outputs generated by BEATRICE. The results are output in (1) a PDF document that displays the PIPs and corresponding credible sets, (2) a table with PIPs, (3) a text file with credible sets, and (4) a text file with the conditional inclusion probability of the variants within the credible sets. The user can also generate the neural network losses describe in Eq. (12) by adding a flag to the run command.

## 8 Conclusion

We present BEATRICE, a novel Bayesian framework for fine-mapping that identifies potentially causal variants within GWAS risk loci through the shared LD structure. Using a variational approach, we approximate the posterior probability of the causal location(s) via a binary concrete distribution. We leverage the unique properties of binary concrete random variables to build an optimization algorithm that can successfully model variants with differing levels of association. Moreover, we introduce a new strategy to build a reduced set of causal configurations within the exponential search space that can be neatly folded into our optimization routine. This reduced set is used to approximate the PIPs and identify credible sets. In a detailed simulation study, we compared BEATRICE with two state-of-the-art baselines and demonstrated the advantages of BEATRICE under different noise settings. Finally, our model does not have any prior on the causal variants and is agnostic to the original GWAS study. Hence, BEATRICE is a powerful tool to refine the results of a GWAS or eQTL analysis. It is also flexible enough to accommodate a variety of experimental settings.

## Supplementary Material

Supplement 1
