# Supplementary material for: BEATRICE: Bayesian Fine-mapping from Summary Data using Deep Variational Inference
Source: bioRxiv. 2023 Dec 14:2023.03.24.534116. Originally published 2023 Mar 25. Preprint. [Version 2] doi: 10.1101/2023.03.24.534116 (PMC10055416; doi:10.1101/2023.03.24.534116)
Supplement: Supplementary file 1 [file media-1.pdf]

Supplement for BEATRICE: Bayesian Fine-mapping from Summary Data using Deep Variational Inference

Sayan Ghosal[1], Michael C. Schatz[2], Archana Venkataraman[1,3*]

1 Department of Electrical and Computer Engineering, Johns Hopkins University, Baltimore, MD, USA
2 Department of Computer Science, Johns Hopkins University, Baltimore, MD, USA
3 Department of Electrical and Computer Engineering, Boston University, Boston, MA, USA

* archanav@bu.edu

S1 Detailed Methods and Additional Applications

S1.1 Distribution of Normalized GWAS Effect Sizes

GWAS uses a collection of element-wise linear regression models to estimate the effect of each genetic variant. Mathematically, the collection of GWAS effect sizes are computed as $\hat{\beta} = \frac{1}{n}\mathbf{X}^T\mathbf{y}$, with the corresponding vector of normalized z-scores equal to $\mathbf{z} = \sqrt{\frac{\tau}{n}}\mathbf{X}^T\mathbf{y}$ [1,2]. Under additive linear assumption of the quantitative trait (Section 2.1), we can write the normalized effect sizes as follows:

$$\mathbf{z} = \sqrt{\frac{\tau}{n}}\mathbf{X}^T\left(\mathbf{X}\beta + \eta\right) \text{ where } \eta \sim N\left(0, \frac{1}{\tau}\mathbf{I}_n\right) \tag{1}$$

$$\implies P\left(\mathbf{z}|\beta\right) = N\left(\mathbf{z}; \sqrt{(n\tau)}\beta\mathbf{\Sigma}_X, \mathbf{\Sigma}_X\right) \tag{2}$$

Under the assumptions of our generative process, the prior on the effect sizes β follows a normal distribution $N\left(0, \frac{1}{\tau}\sigma^2\mathbf{\Sigma}_C\right)$. Under this assumed prior, the posterior distribution of the z-scores $\mathbf{z}$ can be written as follows:

$$p\left(z|\mathbf{\Sigma}_X, \mathbf{\Sigma}_C\right) = N\left(\mathbf{z}; 0, \mathbf{\Sigma}_X + \mathbf{\Sigma}_X\left(n\sigma^2\mathbf{\Sigma}_C\right)\mathbf{\Sigma}_X\right) \tag{3}$$

S1.2 Properties of Binary Concrete Random Vectors

Each element of a binary concrete random vector can be viewed as a continuous relaxation of a Bernoulli random variable that can be mathematically represented as

$$\mathbf{c}_i = \xi\left(\frac{\log\left(\frac{U}{1-U}\right) + \log\left(\frac{\mathbf{p}_i}{1-\mathbf{p}_i}\right)}{\lambda}\right), \tag{4}$$

where $\xi(\cdot)$ is a sigmoid function, λ controls the extent of relaxation from Bernoulli random variable, U is a uniform random variable that introduces randomness, and $\mathbf{p}_i$ is an approximate probability measure of finding a causal variant at location i. As shown in Figure S1(a) smaller values of λ lead to progressively discretized $\mathbf{c}_i$, while larger values provide a smoother mapping. This continuous representation allows us to model

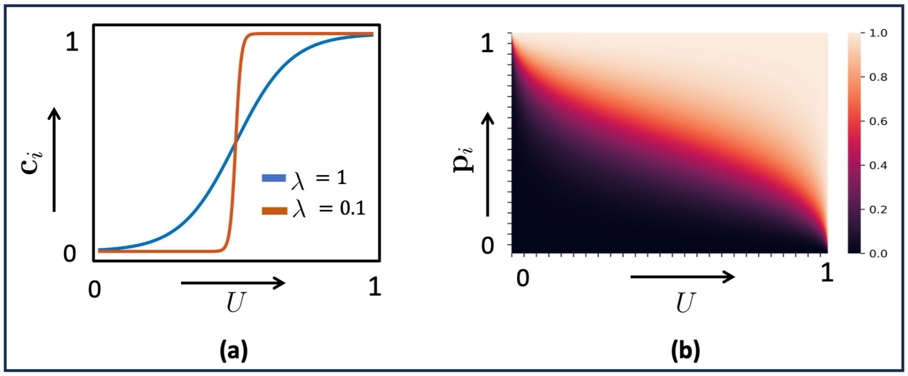

Fig S1. Properties of the binary concrete distribution. (a) Relationship between $\mathbf{c}_i$ and U for different values of λ. (b) The change in $\mathbf{c}_i$ for varying probability map value $\mathbf{p}_i$ and uniform noise U. The darker and brighter colors represents $\mathbf{c}_i$ close to 0 and 1, respectively.

the infinitesimal effects of the non-causal variants. In Figure S1(b) we show how the joint relationship between $\mathbf{p}_i$ and the sampled uniform random variable U generates the binary concrete random variable $\mathbf{c}_i$. As seen, higher values of $\mathbf{p}_i$ push $\mathbf{c}_i$ closer to 1, irrespective of the uniform random variable. Intuitively, a variant with high probability is a likely candidate to contain a causal variant.

S1.3 Identification of Credible Sets for BEATRICE

One of the notable features of BEATRICE is its ability to identify a comprehensive set of causal configurations with non-negligible posterior probability within the exponentially large search space. As described in Section 3.1, the reduced search space $\mathcal{B}^R$ is comprised of vectors that BEATRICE randomly samples at each iteration of the optimization. We identify credible sets from $\mathcal{B}^R$ in two steps.

First, in a sequential fashion, we identify the "key" variant with the highest conditional probability given the previously selected variants. Formally, let $\mathcal{K}$ be the indices of previously identified "key" variants. The conditional probability for variant i given $\mathcal{K}$ in each iteration can be calculated as follows:

$$P\left(\mathbf{b}_i = 1|\mathcal{K}, \mathbf{z}, \mathbf{\Sigma}_X\right) = \frac{\sum_{\mathbf{b}\in\mathcal{C}} P\left(\mathbf{b}|\mathbf{z}, \mathbf{\Sigma}_X\right)}{\sum_{\mathbf{b}'\in\mathcal{D}} P\left(\mathbf{b}'|\mathbf{z}, \mathbf{\Sigma}_X\right)} \tag{5}$$

$$\text{s.t. } \mathcal{D} \subset \mathcal{B}^R \text{ and } \mathcal{D} = \{\mathbf{b}|\mathbf{b}_j = 1 \;\forall\; j \in \mathcal{K}\}$$
$$\mathcal{C} \subset \mathcal{B}^R \text{ and } \mathcal{C} = \{\mathbf{b}|\mathbf{b}_j = 1 \;\forall\; j \in \{i\} \cup \mathcal{K}\}$$

where, $\mathcal{D}$ is the subset of $\mathcal{B}^R$ that includes all of "key" variants and $\mathcal{C}$ is the subset of $\mathcal{B}^R$ that includes both variant i and the "key" variants.

We perform this sequential variant selection until the maximum posterior probability reduces below a threshold, which we define as the "key" threshold γ_{key} and fix at $\gamma_{key} = 0.2$ for all experiments. We note that this threshold can be controlled by the user. The selected "key" variants act as proxy for highly plausible causal variants.

In the second step, we identify the set of variants that can replace the "key" variant in the causal configurations while maintaining a high posterior probability. This set of variants act as a credible set for that particular "key" variant. To do this, we first remove one of the key variants from $\mathcal{K}$ and estimate the posterior probability of other variants given the remaining "key" variants. For example, let variant k_1 be a " key"

Algorithm 1 Algorithm to find credible sets

$\mathcal{K} = \{\}$
$\mathcal{CS} = \{\}$
Estimate posterior probabilities accoding to Eq. 5.
while $max\,[P(\mathbf{b}_i|\mathcal{K},\mathbf{z},\mathbf{\Sigma}_X) \mid \forall i \notin \mathcal{K}] > \gamma_{key}$ **do**
    $\mathcal{K} = \mathcal{K} \cup argmax\,[P(\mathbf{b}_i|\mathcal{K},\mathbf{z},\mathbf{\Sigma})_X \mid \forall i \notin \mathcal{K}]$
    Estimate posterior probabilities accoding to Eq. 5.
end while
for $k \in \mathcal{K}$ **do**
    $\mathcal{S} = \{\}$
    $cov = 0$
    Generate $\mathcal{K}'$ by removing k for "key" set.
    for $i = [1, \ldots, m]$ and $i \notin \mathcal{K}'$ **do**
        if allow_dup == False **then**
            Remove i-th SNPs $\forall i \in \mathcal{CS}$
        end if
        Estimate posterior probability according to Eq. 6
        Stack the probability scores in a vector $\mathcal{P}$.
    end for
    while $max\,[\mathcal{P}_i \mid \forall i \notin \mathcal{K}'] > \gamma_{selection}$ and $cov < \gamma_{coverage}$ **do**
        $\mathcal{S} = \mathcal{S} \cup argmax\,[\mathcal{P}_i \mid \forall i \notin \mathcal{K}']$
        $cov = cov + max\,[\mathcal{P}_i \mid \forall i \notin \mathcal{K}']$
    end while
    Add $\mathcal{S}$ to $\mathcal{CS}$ as credible set of k.
end for

variant. We estimate the posterior probabilities as follows:

$$P\left(\mathbf{b}_i = 1|\mathcal{K}', \mathbf{z}, \mathbf{\Sigma}_X\right) = \frac{\sum_{\mathbf{b}\in\mathcal{G}} P\left(\mathbf{b}|\mathbf{z}, \mathbf{\Sigma}_X\right)}{\sum_{\mathbf{b}'\in\mathcal{H}} P\left(\mathbf{b}'|\mathbf{z}, \mathbf{\Sigma}_X\right)} \tag{6}$$

$$\text{s.t. } \mathcal{K} = \mathcal{K}' \cup \{k_1\}$$
$$\mathcal{G} = \{\mathbf{b}|\mathbf{b}_j = 1 \ \forall\ j \in \{i\} \cup \mathcal{K}'\}$$
$$\mathcal{H} \subset \mathcal{B}^R \text{ and } \mathcal{H} = \{\mathbf{b}|\mathbf{b}_j = 1 \ \forall\ j \in \mathcal{K}'\}$$

where $\mathcal{K}'$ is the set of "key" variants without k_1, $\mathcal{G}$ is the set of configurations that include both variant i and the remaining "key" variants, and and $\mathcal{H}$ is the set of causal configurations that include all "key" variants except k_1.

Once computed, we sort these posterior probabilities in descending order and add the corresponding variants to the credible set until the cumulative sum reaches the coverage threshold $\gamma_{coverage}$. By default, we do not allow overlap between the credible sets. However, this setting can be relaxed using the flag *allow_dup* when calling BEATRICE. We fix the coverage threshold at $\gamma_{key} = 0.95$ in this work, but it too can be set by the user. Finally, we prune uncorrelated variants by thresholding the posterior probability according to the selection threshold $\gamma_{selection} = 0.05$, again a tunable parameter for users. Algorithm 1 provides a detailed description of these steps.

S1.4 Detailed Architecture of Inference Module

BEATRICE consists of three main components: an inference module, a random sampler, and a generative module. Figure S2 shows the detailed neural network architecture of

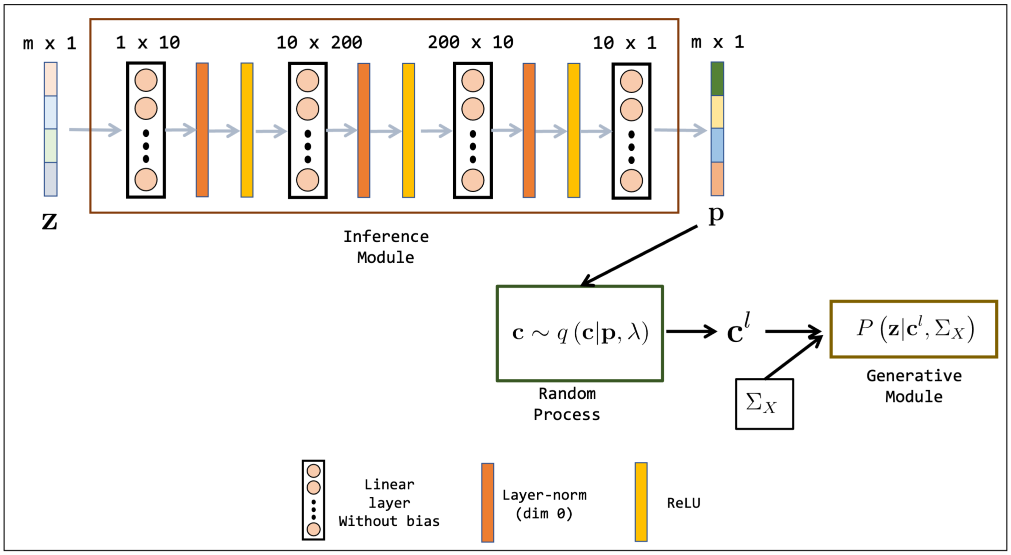

Fig S2. Neural network architecture for the inference module used in BEATRICE. The neural network uses a sequence of linear layers, layer normalization, and activation layers. The dimension of the linear layers are shown on top of each layer. The input to the inference module is the normalized z-scores obtained from GWAS. The output of the inference module is the estimated parameters of our binary concrete distribution.

the inference module. The neural network is trained to output the parameters $\mathbf{p}$ of the binary concrete distribution that we use to approximate the posterior distribution of the causal configurations given the z-statistics and LD matrix. We use the variable ϕ to denote the collection of learnable weights in the neural network. The weights ϕ are trained using gradient descent to minimize the KL divergence loss given in Eq. (9) of the manuscript. This process goes as follows: given an an input z-statistic (1) the neural network generates parameters $\mathbf{p}(\theta)$; (2) the random process sampler uses these parameters to generate the causal configuration $\mathbf{c}$ according to Eq. (6); (3) the generative module uses Eq. (4) to compute the log-likelihood. Finally, we compute the KL divergence loss and use gradient descent to update the neural network weights ϕ. This three-step process is repeated until convergence. Notice that ϕ is a function of $\mathbf{z}$ and $\mathbf{\Sigma}_X$ because the KL divergence loss is a function of both these inputs. This property ensures that the neural network uses the data to generate optimal parameters $\mathbf{p}$ for our proposal distribution $q(\cdot; \mathbf{p}, \lambda)$. The implicit function learned by this network helps BEATRICE to handle complex and possibly nonlinear interactions in the data without being constrained by parametric representations.

S1.5 Identification of Credible Sets for FINEMAP

FINEMAP outputs a collection of credible sets under the assumption of multiple causal variants $d = 1, \ldots, D$. Similar to the approach used in [3], we sub-select the credible sets from this collection with the highest posterior probability. From here, we pruned the sets with minimum absolute purity greater that 0.5. As defined in [3], purity is the pairwise correlation coefficient between the variants, obtained from the LD matrix.

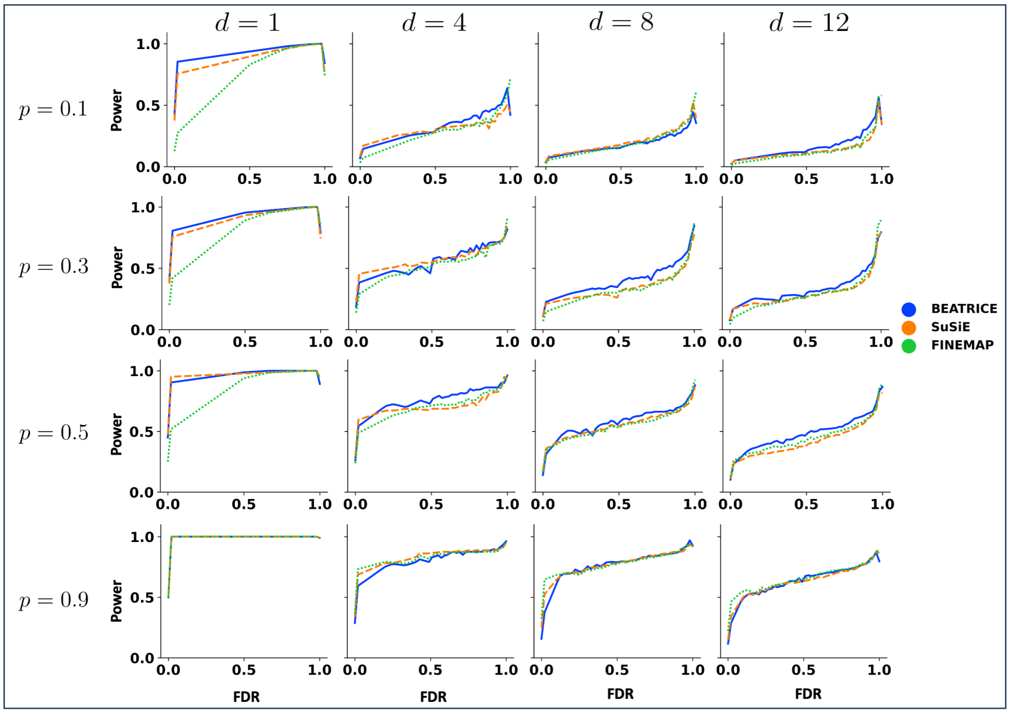

Fig S3. Power vs. FDR curve for three models across multiple causal variants $d = [1, 4, 8, 12]$, and multiple proportion of phenotype variance explained by causal variants $p = [0.1, 0.3, 0.5, 0.9]$, while fixing SNP heritability at $\omega^2 = 0.4$. Each row and column corresponds to a specific value of p and d, respectively. In each plot, the y-axis captures power, and the x-axis represents FDR.

S1.6 Additional FDR vs. Power Curves for Different Values of ω^2

Figure S3 and Figure S4 show the FDR vs. power performance across models for SNP heritability $\omega^2 = 0.4$ and $\omega^2 = 0.8$, respectively. We again notice that in the presence of non-zero infinitesimal effects, BEATRICE gives the best performance. This property shows BEATRICE is consistent across different SNP heritability values. These results suggest that BEATRICE can better estimate the causal variant(s) in the presence of confounding information from non-causal variants.

S1.7 Detailed Comparison Analyses

In this section, we compare the performances of the models across individual noise settings and provide further insight about the advantages of BEATRICE. Figure S6, Figure S7, and Figure S5 show the performance comparison of AUPRC, power and coverage, respectively. Figure S5 shows a significant improvement in coverage compared to the baselines across noise settings. In addition, the BEATRICE shows uniformly better AUPRC in Figure S6. However, in terms of power, all models exhibit similar performance. A high coverage with comparable power suggests that BEATRICE can identify high quality credible sets that contain causal variants. In contrast, the baselines identify many credible that do not contain a causal variant, ultimately leading to low coverage.

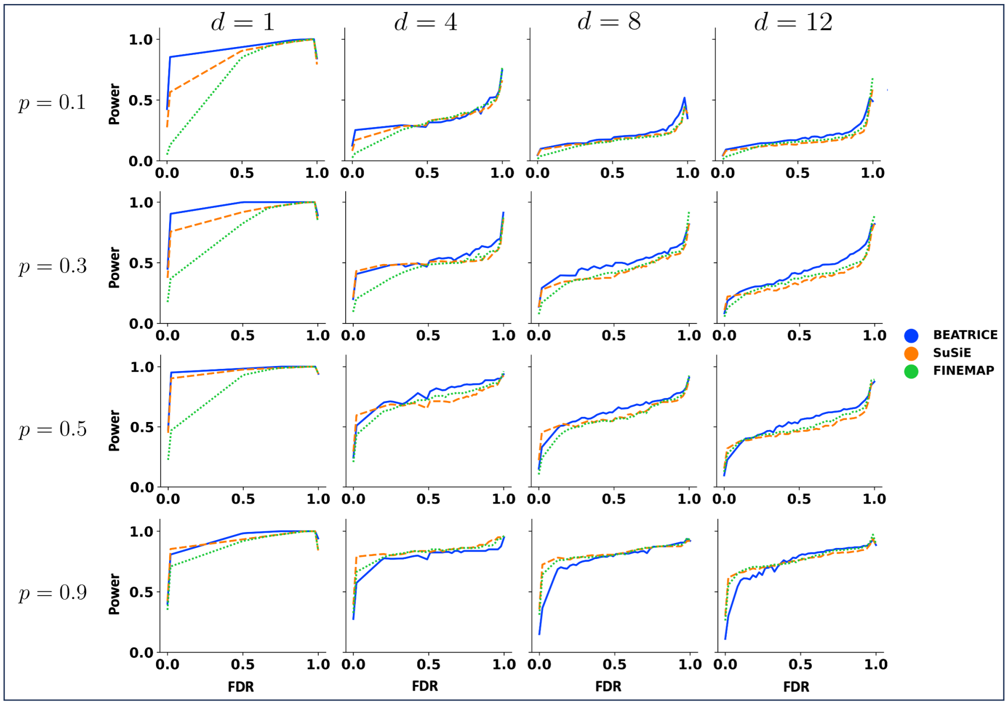

Fig S4. Power vs. FDR curve for three models across multiple causal variants $d = [1, 4, 8, 12]$, and multiple proportion of phenotype variance explained by causal variants $p = [0.1, 0.3, 0.5, 0.9]$, while fixing SNP heritability at $\omega^2 = 0.8$. Each row and column corresponds to a specific value of p and d, respectively. In each plot, the y-axis captures power, and the x-axis represents FDR.

S1.8 Performance Comparison When Allowing Overlap in the Credible Sets

In this Section, we compare the performance of BEATRICE , FINEMAP, and SuSiE when allowing for overlap between credible sets. In the case of BEATRICE , we enable this setting through a flag in the function call. In contrast, SuSiE and FINEMAP do not have an explicit flag to control the credible sets. Nonetheless, we notice empirically that among 2400 simulation experiments, there are 21 and 29 scenarios where FINEMAP, and SuSiE, have overlapping variants across credible sets. Such scenarios can arise when two credible sets, estimated for different causal variants, contain other variants with high LD. Similar to Section 4.3.1, we report the AUPRC, coverage, power, and size while sweeping over the number of causal variants d, the genotype variance ω^2, and the percentage of phenotypic variance explained by the causal SNPs p.

Figure S8, Figure S9, and Figure S10 report the performance of each model for different values of d, ω^2, and p, respectively. As seen, BEATRICE has slightly lower power than when enforcing non-overlapping credible sets (main text), but it remains within the 95% confidence interval of both SuSiE and FINEMAP. This slight difference occurs because SuSiE and FINEMAP generate a larger number of credible sets as compared to BEATRICE, with many of them not containing a causal variant. This scenario allows SuSiE and FINEMAP to cover many variants, improving power. At the same time, the coverage of these methods is much lower. The trends in AUPRC and credible set size are similar to the case of non-overlapping sets.

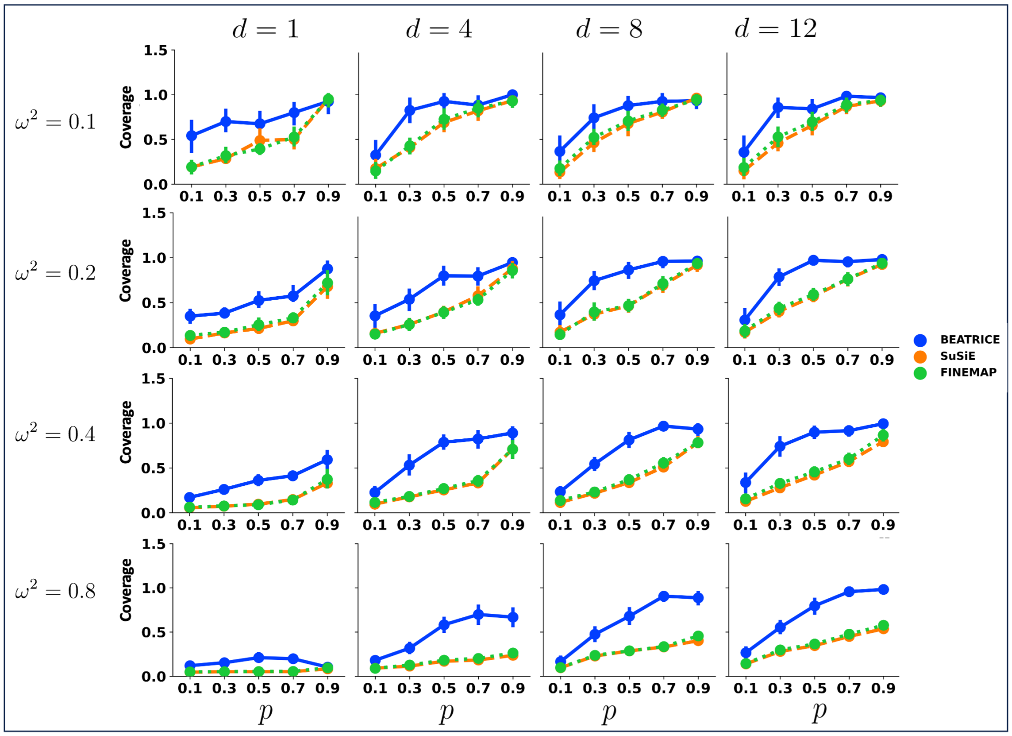

Fig S5. Coverage of the credible sets generated by the three models across multiple causal variants $d = [1, 4, 8, 12]$, multiple SNP heritability $\omega^2 = [0.1, 0.2, 0.4, 0.8]$ and multiple infinitesimal effects from non-causal variants $p = [0.1, 0.3, 0.5, 0.7, 0.9]$. Each row and column corresponds to a specific value of ω^2 and d, respectively. In each plot, the y-axis captures coverage, and the x-axis represents p.

S1.9 Comparison with SuSiE-inf

SuSiE-inf [4] is an extension of the SuSiE model that accounts for infinitesimal effects from non-causal variants. In this section, we compare the performance of BEATRICE with SuSiE-inf across the same simulation setting as in Section 4.1 of the main text.

Unlike BEATRICE, we observe that SuSiE-inf fails to converge in multiple cases. Specifically, Figure S11 illustrates the number of experimental settings, for which SuSie-inf fails to converge across each parameter sweep. This problem becomes prominent with increasing SNP heritability, as explained by ω^2. We believe that the SuSiE-inf algorithm, as described in the preprint [4], is still in development with multiple issues with numerical stability.

Figure S12, Figure S13, and Figure S14 shows the performance comparison between BEATRICE and SuSiE-inf. We emphasize that the performance of SuSiE-inf is computed based only on the convergent runs, so these values should be treated as optimistic. In contrast, the performance of BEATRICE is computed across all runs, as we did not face convergence issues with our model. Across different parameter sweeps, we see that the coverage of SuSiE-inf is similar to BEATRICE. However, BEATRICE achieves uniformly better power and AUPRC.

S1.10 Hyper-parameters of the Baseline Methods

We compare BEATRICE with SuSiE-v0.12.27 and FINEMAP-v1.4.1.

During the implementation of SuSiE, we provide the un-normalized effect sizes (β),

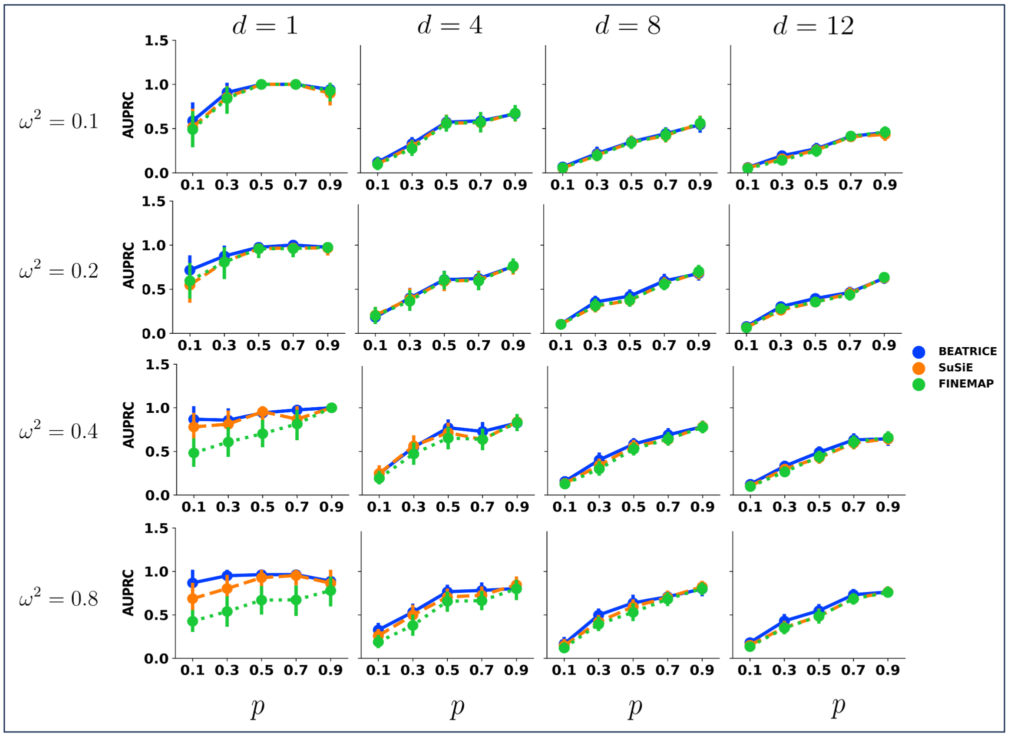

Fig S6. AUPRC of PIPs generated by the three models across multiple causal variants $d = [1, 4, 8, 12]$, multiple SNP heritability $\omega^2 = [0.1, 0.2, 0.4, 0.8]$ and multiple infinitesimal effects from non-causal variants $p = [0.1, 0.3, 0.5, 0.7, 0.9]$. Each row and column corresponds to a specific value of ω^2 and d, respectively. In each plot, the y-axis captures AUPRC, and the x-axis represents p.

the Standard Error (SE) of the effect sizes, the LD matrix, the phenotype variance, and the number of samples. Additionally, we fix the number of causal variants to 20 and we estimate the residual variance. The rest of the hyperparameters are fixed to default values, as described in
https://stephenslab.github.io/susieR/articles/finemapping_summary_statistics.html.

We implement FINEMAP using the stochastic shotgun approach. During implementation, we fix the number of causal variants to 20 and the rest of the hyperparameters are fixed to default values, as described in
http://christianbenner.com/

References

1. Uffelmann E, Huang QQ, Munung NS, de Vries J, Okada Y, Martin AR, et al. Genome-wide association studies. Nature Reviews Methods Primers 2021 1:1. 2021;1:1–21. doi:10.1038/s43586-021-00056-9.

2. Chen W, Larrabee BR, Ovsyannikova IG, Kennedy RB, Haralambieva IH, Poland GA, et al. Fine mapping causal variants with an approximate bayesian method using marginal test statistics. Genetics. 2015;200(3):719–736.

3. Zou Y, Carbonetto P, Wang G, Stephens M. Fine-mapping from summary data with the "Sum of Single Effects" model. PLOS Genetics. 2022;18:e1010299. doi:10.1371/JOURNAL.PGEN.1010299.

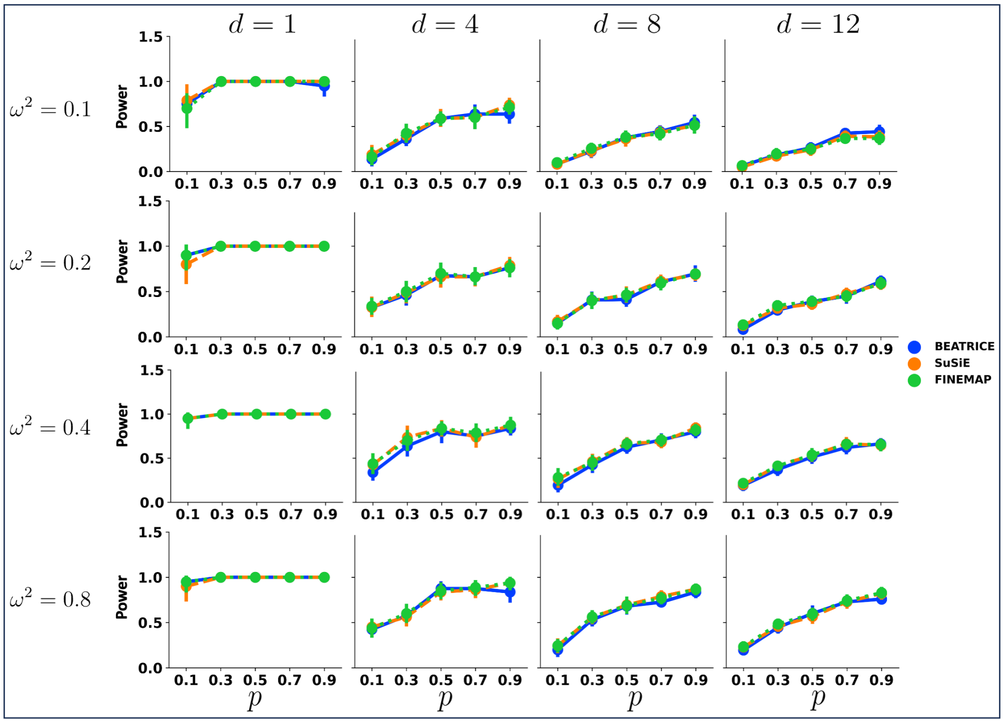

Fig S7. Power of the credible sets generated by three models across multiple causal variants $d = [1, 4, 8, 12]$, multiple SNP heritability $\omega^2 = [0.1, 0.2, 0.4, 0.8]$ and multiple infinitesimal effects from non-causal variants $p = [0.1, 0.3, 0.5, 0.7, 0.9]$. Each row and column corresponds to a specific value of ω^2 and d, respectively. In each plot, the y-axis captures power, and the x-axis represents p.

4. Cui R, et al. Improving fine-mapping by modeling infinitesimal effects. bioRxiv. 2022;doi:10.1101/2022.10.21.513123.

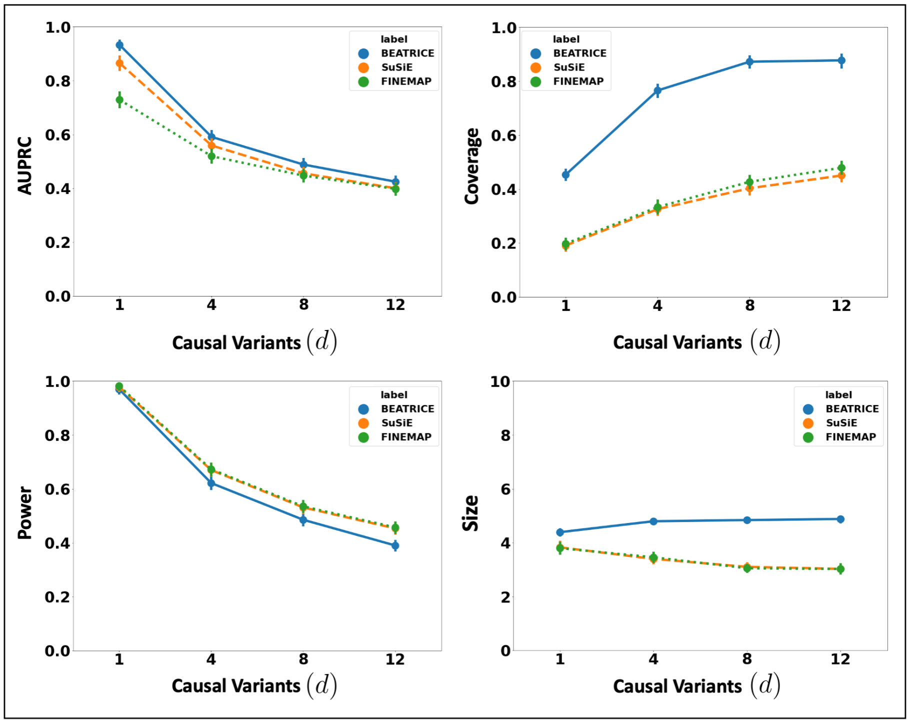

Fig S8. The performance metrics for the three methods across varying numbers of causal variants. Along the x-axis, we plot the number of causal variants, and across the y-axis, we plot the mean and confidence interval (95%) of each metric. We calculate the mean by fixing d to a specific value $d = d^*$ and sweep over all the noise settings where $d = d^*$.

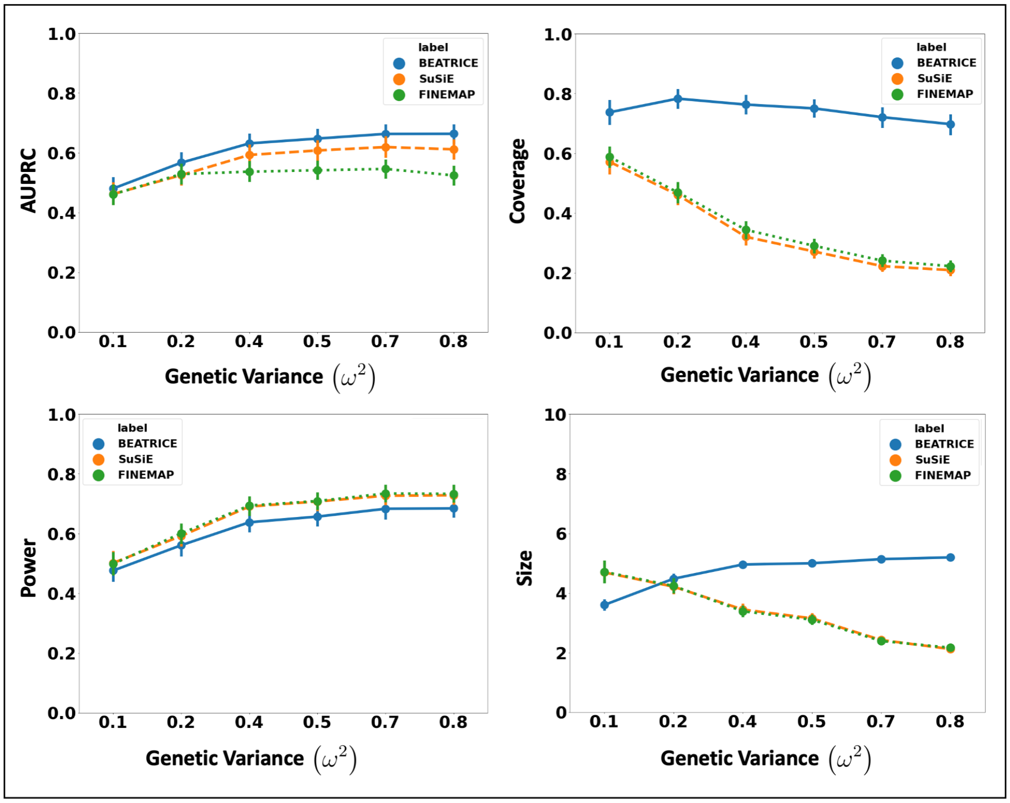

Fig S9. The performance metric for increasing phenotype variance explained by genetics. Along the x-axis, we plot the variance explained by genetics (ω^2), and across the y-axis, we plot each metric's mean and confidence interval (95%). We calculate the mean by fixing ω^2 to a specific value $\omega = \omega^*$ and sweep over all the noise settings where $\omega = \omega^*$.

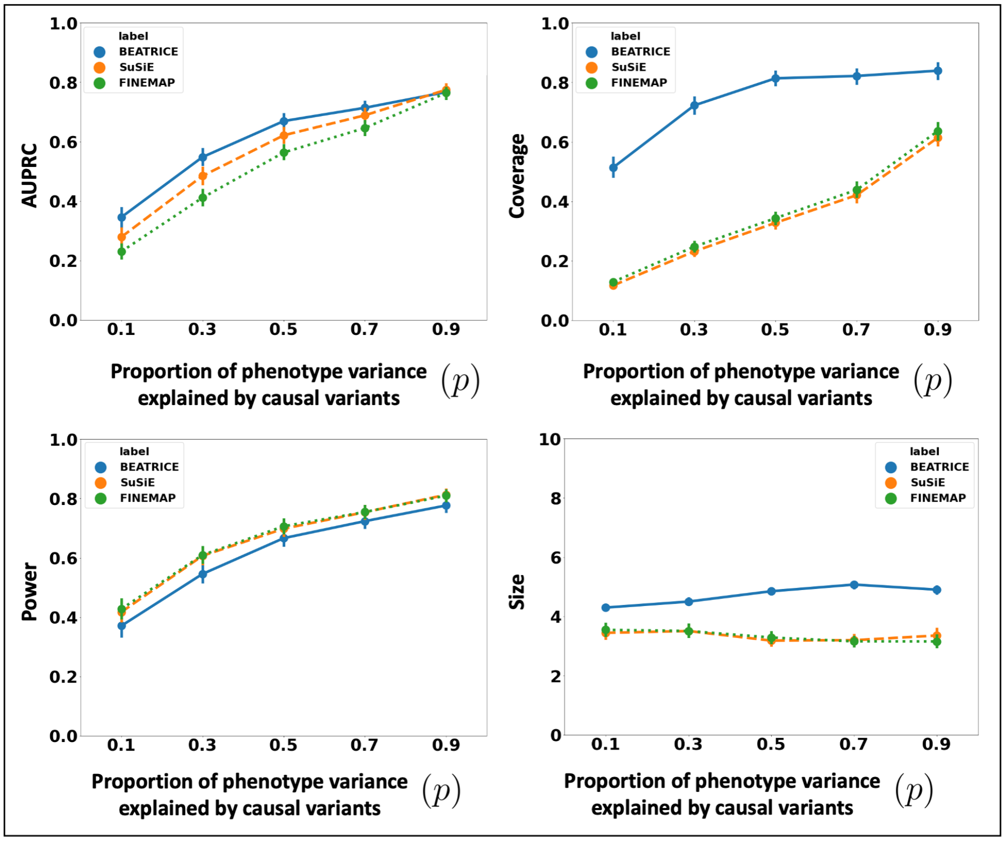

Fig S10. The performance metric for multiple levels of noise introduced by non-causal variants. The noise level (p) is explained by the variance ratio of non-causal variants vs. causal variants. Along the x-axis, we plot the noise level (p); across the y-axis, we plot each metric's mean and confidence interval (95%). We calculate the mean by fixing p to a specific value $p = p^*$ and sweep over all the noise settings where $p = p^*$.

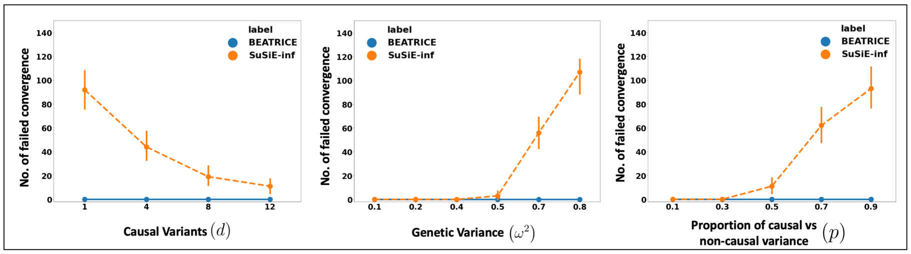

Fig S11. Number of non-convergent runs of SuSiE-inf, as compared to BEATRICE.

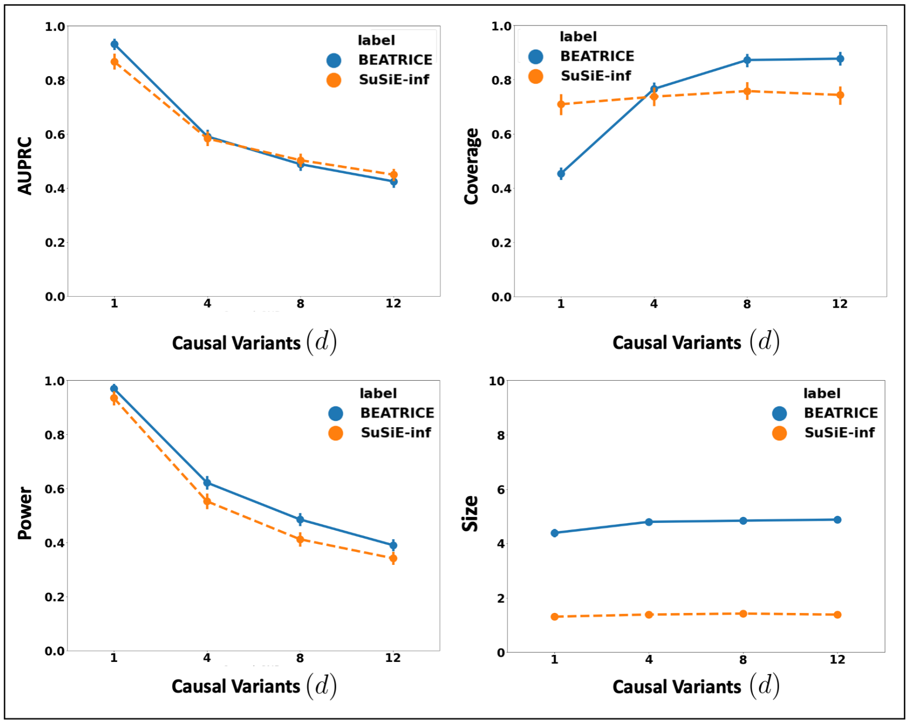

Fig S12. Performance metrics of BEATRICE and SuSiE-inf across varying numbers of causal variants. The performance of SuSiE-inf is calculated over the subset of simulation settings in which the algorithm converges; non-convergent settings are omitted from the analysis. The x-axis corresponds to the number of causal variants, and the y-axis plots the mean and confidence interval (95%) of each metric. We calculate the mean by fixing d to a value $d = d^*$ and sweeping over all the noise settings where $d = d^*$.

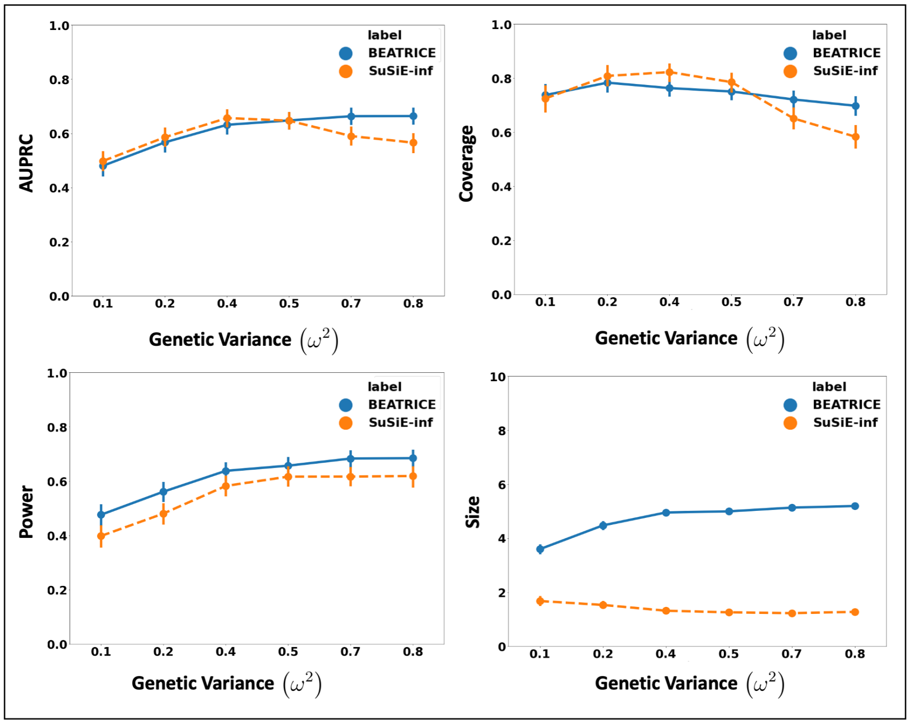

Fig S13. Performance metrics of BEATRICE and SuSiE-inf for increasing phenotype variance explained by genetics. The performance of SuSiE-inf is calculated over the subset of simulation settings in which the algorithm converges; non-convergent settings are omitted from the analysis. The x-axis corresponds to the variance explained by genetics (ω^2), and the y-axis plots the mean and confidence interval (95%) of each metric. We calculate the mean by fixing ω^2 to a value $\omega = \omega^*$ and sweeping over all the noise settings where $\omega = \omega^*$.

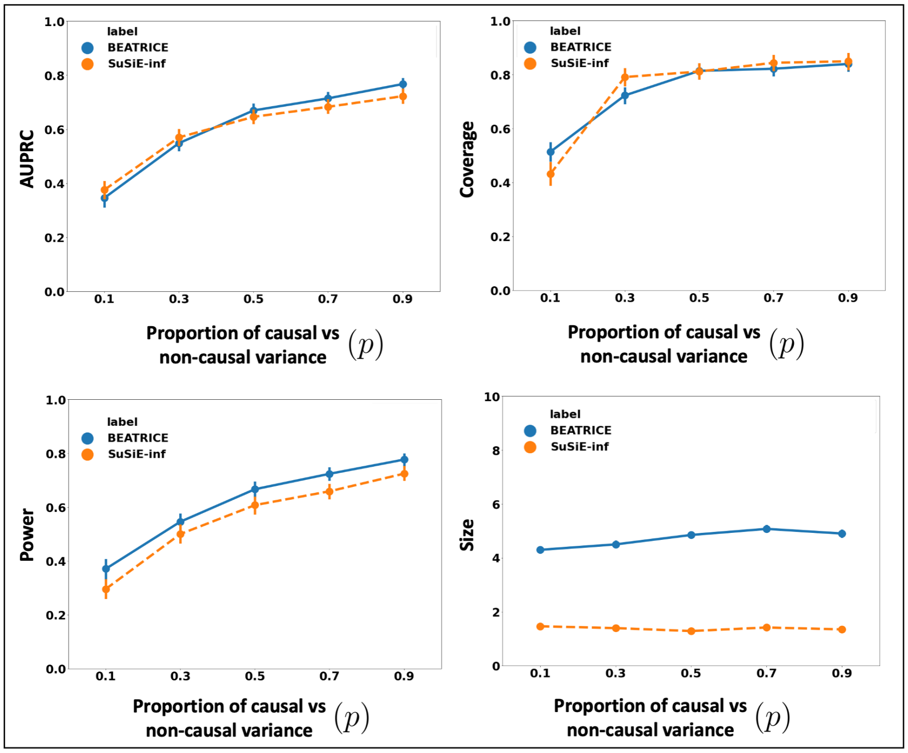

Fig S14. Performance metrics of BEATRICE and SuSiE-inf for multiple levels of noise introduced by non-causal variants. The performance of SuSiE-inf is calculated over the subset of simulation settings in which the algorithm converges; non-convergent settings are omitted from the analysis. The x-axis corresponds to the noise level (p), and the y-axis plots the mean and confidence interval (95%) of each metric. We calculate the mean by fixing p to a value $p = p^*$ and sweeping over all the noise settings where $p = p^*$.
