# Supplementary material for: BEATRICE: Bayesian Fine-mapping from Summary Data using Deep Variational Inference
Source: bioRxiv. 2023 Mar 25:2023.03.24.534116. Preprint. [Version 1] doi: 10.1101/2023.03.24.534116 (PMC10055416; doi:10.1101/2023.03.24.534116)
Supplement: Supplementary file 1 [file media-1.pdf]

Supplement for BEATRICE: Bayesian Fine-mapping from Summary Data using Deep Variational Inference

Sayan Ghosal[1], Michael C. Schatz[2], Archana Venkataraman[1,3*]

1 Department of Electrical and Computer Engineering, Johns Hopkins University, Baltimore, MD, USA
2 Department of Computer Science, Johns Hopkins University, Baltimore, MD, USA
3 Department of Electrical and Computer Engineering, Boston University, Boston, MA, USA

* archanav@bu.edu

1 S1 Distribution of Normalized GWAS Effect Sizes

GWAS uses a collection of element-wise linear regression models to estimate the effect of each genetic variant. Mathematically, the collection of GWAS effect sizes are computed as $\hat{\beta} = \frac{1}{n}\mathbf{X}^T\mathbf{y}$, with the corresponding vector of normalized z-scores equal to $\mathbf{z} = \sqrt{\frac{\tau}{n}}\mathbf{X}^T\mathbf{y}$ [1, 2]. Under additive linear assumption of the quantitative trait (Section 2), we can write the normalized effect sizes as follows:

$$\mathbf{z} = \sqrt{\frac{\tau}{n}}\mathbf{X}^T\left(\mathbf{X}\beta + \eta\right) \text{ where } \eta \sim N\left(0, \frac{1}{\tau}\mathbf{I}_n\right) \tag{1}$$

$$\implies P\left(\mathbf{z}|\beta\right) = N\left(\mathbf{z}; 0, (n\tau)\mathbf{\Sigma}_X\beta\mathbf{\Sigma}_X + \mathbf{\Sigma}_X\right) \tag{2}$$

Under the assumptions of our generative process, the prior on the effect sizes β follows a normal distribution $N\left(0, \frac{1}{\tau}\sigma^2\mathbf{\Sigma}_C\right)$. Under this assumed prior, the posterior distribution of the z-scores $\mathbf{z}$ can be written as follows:

$$p\left(z|\mathbf{\Sigma}_X, \mathbf{\Sigma}_C\right) = N\left(\mathbf{z}; 0, \mathbf{\Sigma}_X + \mathbf{\Sigma}_X\left(n\sigma^2\mathbf{\Sigma}_C\right)\mathbf{\Sigma}_X\right) \tag{3}$$

2 S2 Detailed Architecture of Inference Module

Our BEATRICE framework consists of three main components: an inference module, a random sampler, and a generative module. Figure 1 shows the detailed neural network architecture of the inference module. The implicit function learned by this network helps BEATRICE to handle complex and possibly nonlinear interactions in the data. The inference module estimates the parameters $\mathbf{p}$ of our proposal distribution $q\left(\cdot; \mathbf{p}, \lambda\right)$. The parameters of the inference module are learned using backpropagation during optimization. Following inference, random sampler uses the parameters $\mathbf{p}$ to output potential causal vectors $\mathbf{c}$ according to the given proposal distribution. Finally, the generative module calculates the likelihood of the observed summary statistics.

3 S3 Identification of Credible Sets for BEATRICE

One of the notable features of BEATRICE is its ability to identify a comprehensive set of causal configurations with non-negligible posterior probability within the

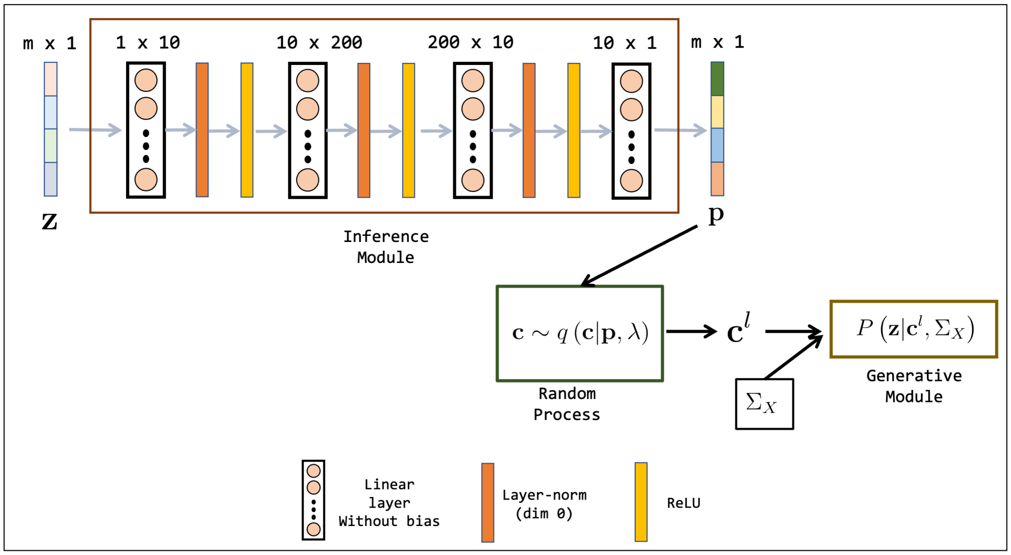

Fig 1. Neural network architecture for the inference module used in BEATRICE. The neural network uses a sequence of linear layers, layer normalization, and activation layers. The dimension of the linear layers are shown on top of each layer. The input to the inference module is the normalized z-scores obtained from GWAS. The output of the inference module is the estimated parameters of our binary concrete distribution.

exponentially large search space. As described in Section 3.5, the reduced search space $\mathcal{B}^R$ is comprised of vectors that BEATRICE randomly samples at each iteration of the optimization. We identify credible sets from $\mathcal{B}^R$ in two steps.

First, in a sequential fashion, we identify the "key" variant with the highest conditional probability given the previously selected variants. Formally, let $\mathcal{K}$ be the indices of previously identified "key" variants. The conditional probability for variant i given $\mathcal{K}$ in each iteration can be calculated as follows:

$$P\left(\mathbf{b}_i = 1|\mathcal{K}, \mathbf{z}, \mathbf{\Sigma}_X\right) = \frac{\sum_{\mathbf{b}\in\mathcal{C}} P\left(\mathbf{b}|\mathbf{z}, \mathbf{\Sigma}_X\right)}{\sum_{\mathbf{b}'\in\mathcal{D}} P\left(\mathbf{b}'|\mathbf{z}, \mathbf{\Sigma}_X\right)} \tag{4}$$

$$\text{s.t. } \mathcal{D} \subset \mathcal{B}^R \text{ and } \mathcal{D} = \{\mathbf{b}|\mathbf{b}_j = 1 \;\forall\; j \in \mathcal{K}\}$$
$$\mathcal{C} \subset \mathcal{B}^R \text{ and } \mathcal{C} = \{\mathbf{b}|\mathbf{b}_j = 1 \;\forall\; j \in \{i\} \cup \mathcal{K}\}$$

where, $\mathcal{D}$ is the subset of $\mathcal{B}^R$ that includes all of "key" variants and $\mathcal{C}$ is the subset of $\mathcal{B}^R$ that includes both variant i and the "key" variants.

We perform this sequential variant selection until the maximum posterior probability reduces below a threshold, which we define as the "key" threshold γ_{key} and fix at $\gamma_{key} = 0.2$ for all experiments. We note that this threshold can be controlled by the user. The selected "key" variants act as proxy for highly plausible causal variants.

In the second step, we identify the set of variants that can replace the "key" variant in the causal configurations while maintaining a high posterior probability. This set of variants act as a credible set for that particular "key" variant. To do this, we first remove one of the key variants from $\mathcal{K}$ and estimate the posterior probability of other variants given the remaining "key" variants. For example, let variant k_1 be a " key"

Algorithm 1 Algorithm to find credible sets

$\mathcal{K} = \{\}$
$\mathcal{CS} = \{\}$
Estimate posterior probabilities accoding to Eq. 4.
while $max\,[P(\mathbf{b}_i|\mathcal{K}, \mathbf{z}, \mathbf{\Sigma}_X) \mid \forall i \notin \mathcal{K}] < \gamma_{key}$ **do**
    $\mathcal{K} = \mathcal{K} \cup argmax\,[P(\mathbf{b}_i|\mathcal{K}, \mathbf{z}, \mathbf{\Sigma})_X \mid \forall i \notin \mathcal{K}]$
    Estimate posterior probabilities accoding to Eq. 4.
end while
for $k \in \mathcal{K}$ **do**
    $\mathcal{S} = \{\}$
    $cov = 0$
    Generate $\mathcal{K}'$ by removing k for "key" set.
    for $i = [1, \ldots, m]$ and $i \notin \mathcal{K}'$ **do**
        Estimate posterior probability according to Eq. 5
        Stack the probabilty scores in a vector $\mathcal{P}$.
    end for
    while $max\,[\mathcal{P}_i \mid \forall i \notin \mathcal{K}'] < \gamma_{selection}$ and $cov < \gamma_{coverage}$ **do**
        $\mathcal{S} = \mathcal{S} \cup argmax\,[\mathcal{P}_i \mid \forall i \notin \mathcal{K}']$
        $cov = cov + max\,[\mathcal{P}_i \mid \forall i \notin \mathcal{K}']$
    end while
    Add $\mathcal{S}$ to $\mathcal{CS}$ as credible set of k.
end for

variant. We estimate the posterior probabilities as follows:

$$P\left(\mathbf{b}_i = 1|\mathcal{K}', \mathbf{z}, \mathbf{\Sigma}_X\right) = \frac{\sum_{\mathbf{b}\in\mathcal{G}} P\left(\mathbf{b}|\mathbf{z}, \mathbf{\Sigma}_X\right)}{\sum_{\mathbf{b}'\in\mathcal{H}} P\left(\mathbf{b}'|\mathbf{z}, \mathbf{\Sigma}_X\right)} \tag{5}$$

$$\begin{aligned} \text{s.t. } \mathcal{K} &= \mathcal{K}' \cup \{k_1\} \\ \mathcal{G} &= \{\mathbf{b}|\mathbf{b}_j = 1 \ \forall \ j \in \{i\} \cup \mathcal{K}'\} \\ \mathcal{H} \subset \mathcal{B}^R \text{ and } \mathcal{H} &= \{\mathbf{b}|\mathbf{b}_j = 1 \ \forall \ j \in \mathcal{K}'\} \end{aligned}$$

where $\mathcal{K}'$ is the set of "key" variants without k_1, $\mathcal{G}$ is the set of configurations that include both variant i and the remaining "key" variants, and and $\mathcal{H}$ is the set of causal configurations that include all "key" variants except k_1.

Once computed, we sort these posterior probabilities in descending order and add the variants to the credible set until the cumulative sum reaches the coverage threshold $\gamma_{coverage}$. We fix the coverage threshold at $\gamma_{key} = 0.95$ in this work, but it too can be set by the user. Finally, we prune uncorrelated variants by thresholding the posterior probability according to the selection threshold $\gamma_{selection} = 0.05$, again a tunable parameter for users. Algorithm 1 provides a detailed description of these steps.

4 S4 Identification of Credible Sets for FINEMAP

FINEMAP outputs a collection of credible sets under the assumption of multiple causal variants $d = 1, \ldots, D$. Similar to the approach used in [3], we sub-select the credible sets from this collection with the highest posterior probability. From here, we pruned the sets with minimum absolute purity greater that 0.5. As defined in [3], purity is the pairwise correlation coefficient between the variants, obtained from the LD matrix.

References

1. Uffelmann E, Huang QQ, Munung NS, de Vries J, Okada Y, Martin AR, et al. Genome-wide association studies. Nature Reviews Methods Primers 2021 1:1. 2021;1:1–21. doi:10.1038/s43586-021-00056-9.

2. Chen W, Larrabee BR, Ovsyannikova IG, Kennedy RB, Haralambieva IH, Poland GA, et al. Fine mapping causal variants with an approximate bayesian method using marginal test statistics. Genetics. 2015;200(3):719–736.

3. Zou Y, Carbonetto P, Wang G, Stephens M. Fine-mapping from summary data with the "Sum of Single Effects" model. PLOS Genetics. 2022;18:e1010299. doi:10.1371/JOURNAL.PGEN.1010299.
